# Supplementary material for: Requirement for PBAF in Transcriptional Repression and Repair at DNA Breaks in Actively Transcribed Regions of Chromatin
Source: Mol Cell. 2014 Sep 4;55(5):723–32. doi: 10.1016/j.molcel.2014.06.028 (PMC4157577; doi:10.1016/j.molcel.2014.06.028)
Supplement: Document S1. Supplemental Experimental Procedures and Figures S1–S6 [file mmc1.pdf]

Molecular Cell, Volume *55*

Supplemental Information

Requirement for PBAF in Transcriptional Repression and Repair at DNA Breaks in Actively Transcribed Regions of Chromatin

Andreas Kakarougkas, Amani Ismail, Anna L. Chambers, Enriqueta Riballo, Alex D. Herbert, Julia Künzel, Markus Löbrich, Penny A. Jeggo, and Jessica A. Downs

SUPPLEMENTAL INFORMATION

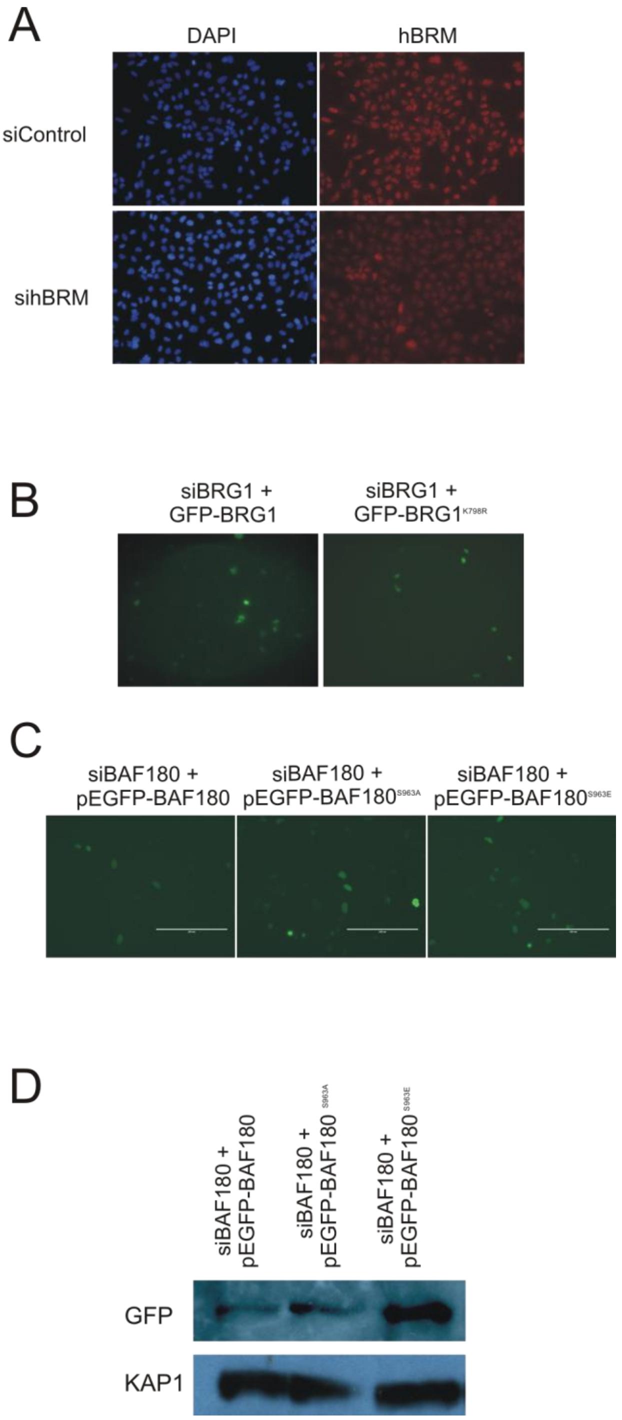

Figure S1, related to Figures 1 and 3. Expression of GFP tagged BAF180 and BRG1 constructs. (A) Knockdown efficiency of hBRM siRNA was assessed by immunofluorescence in A549 cells. (B) Immunofluorescence images showing GFP expression of WT and ATPase mutated GFP-BRG1. Endogenous BRG1 was depleted using siRNA and then the cells were transfected with the siRNA resisant GFP-BRG1 constructs. (C) Immunofluorescence images showing GFP expression of WT, phosphomutant (S963A) and phosphomimic (S963E) GFP-BAF180. Endogenous BAF180 was depleted using siRNA and then the cells were transfected with the siRNA resistant GFP-BAF180 constructs. (D) Expression of the GFP-BAF180 constructs assessed by western blotting using a GFP antibody. KAP1 was used as a loading control.

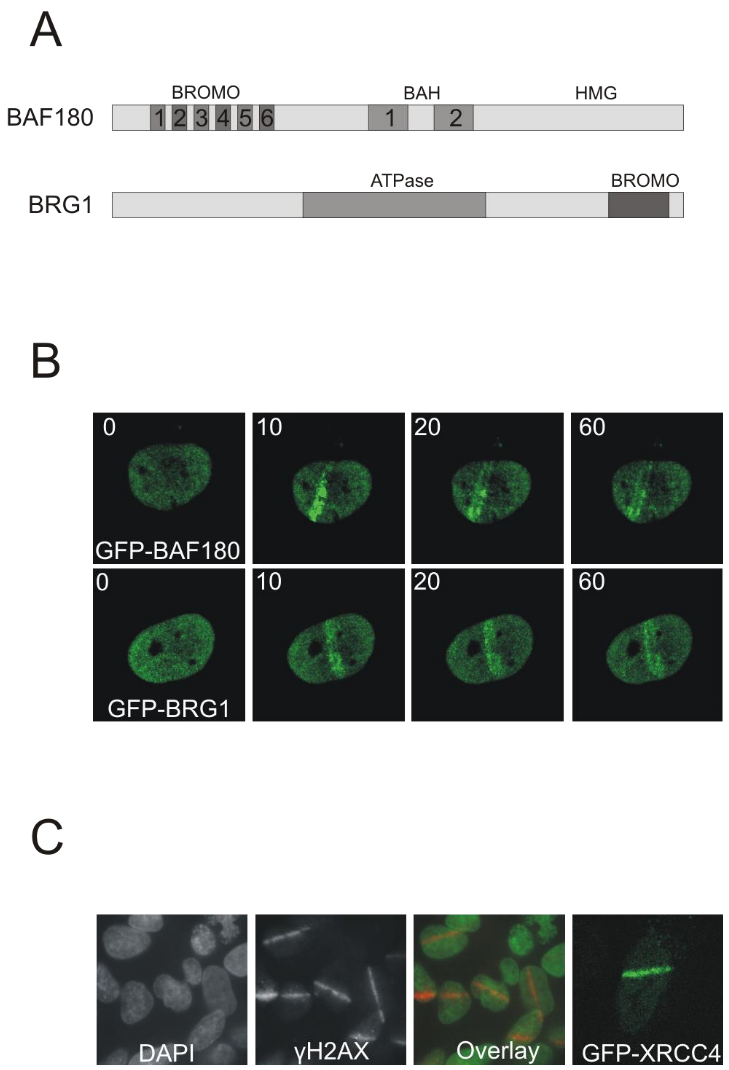

Figure S2, related to Figure 2. BAF180 and BRG1 localise to sites of DNA damage. (A) Schematic representation of BAF180 and BRG1. (B) Localised DNA damage was induced by laser irradiation in cells transfected with EGFP-tagged BAF180 or GFP-tagged BRG1. Representative images up to 60 seconds post laser irradiation. (C) Representative images of γH2AX and GFP-tagged XRCC4 recruitment to sites of laser irradiation, 60 seconds post damage induction.

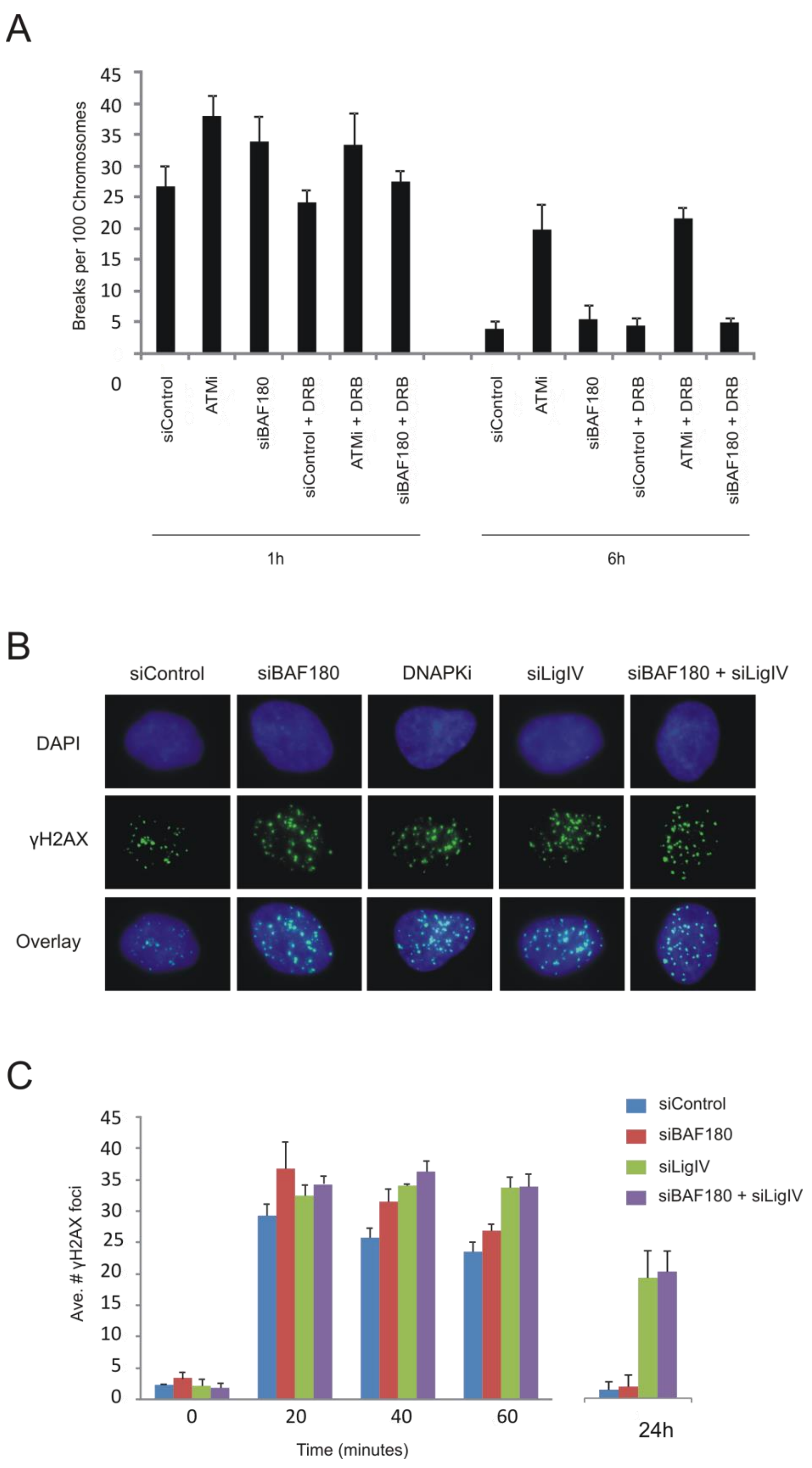

Figure S3, related to Figure 2. BAF180 promotes non-homologous end joining (NHEJ). (A) PCC breakage analysis following 2 Gy IR in HeLa cells treated with the indicated siRNA. Data are represented as mean +/- SD**.** (B) Quantification of γH2AX foci clearance following exposure to 1.5 Gy IR in A549 cells treated with the indicated siRNA. Data are represented as mean +/- SD (C) Representative IF images of A549 cells treated with the indicated siRNA, 40m following exposure to 1.5 Gy IR.

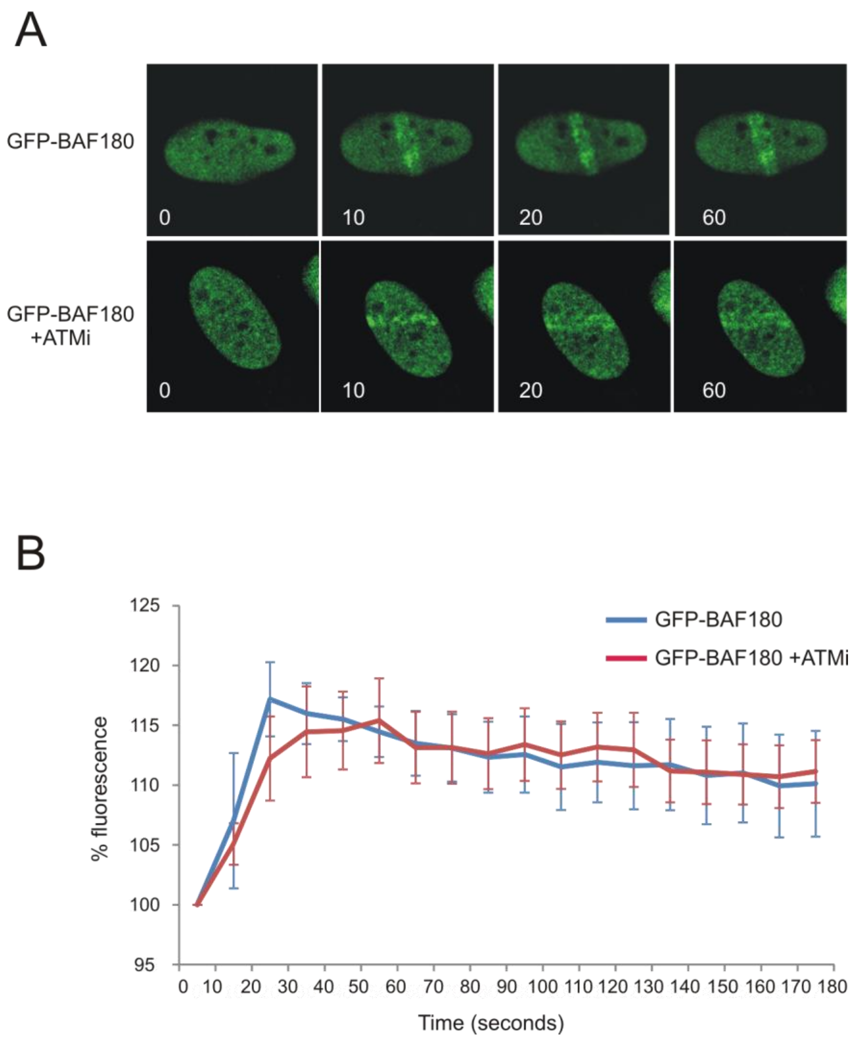

Figure S4, related to Figure 3. BAF180 recruitment to laser induced DNA damage is ATM independent. (A) Localised DNA damage was induced by laser irradiation in cells transfected with GFP-tagged BAF180. In ATMi treated cells, the inhibitor was added 1 hour prior to irradiation. Representative images up to 60 seconds post laser irradiation are shown. (B) Quantification of GFP signal intensity along the laser path in ATMi treated and untreated GFP-BAF180 expressing cells. GFP-signal intensity was monitored for 3 minutes post irradiation. Data are represented as mean +/- SD**.**

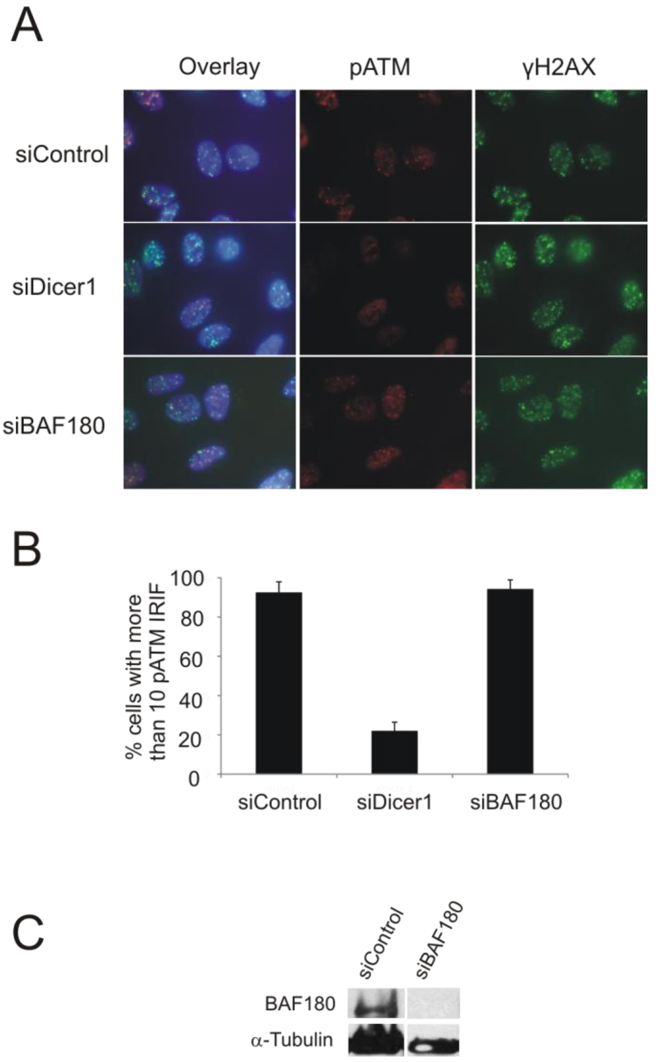

Figure S5, related to Figure 4. BAF180 functions downstream of ATM and is not required for pATM IRIF. (A) pATM and γH2AX IRIF 1 hour after 3 Gy IR in A549 cells treated with the indicated siRNA. (B) Quantification of pATM IRIF. Data are represented as mean +/- SD**.** (C) Western blot analysis of siRNA knockdown efficiency of BAF180 in A549 cells. Tubulin was used as a loading control.

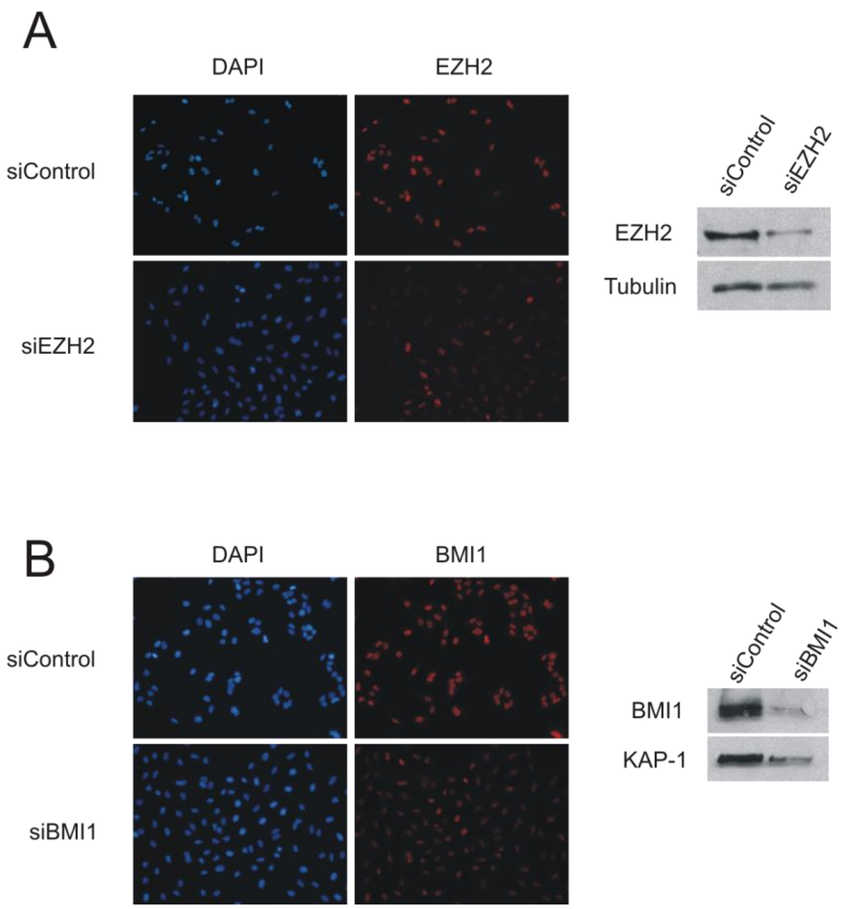

Figure S6, related to Figure 5. Knockdown efficiency of BMI1 and EZH2 siRNA. (A) Knockdown efficiency of BMI1 siRNA was assessed by immunofluorescence and western blotting in A549 cells. KAP1 was used as a loading control. (B) Knockdown efficiency of EZH2 siRNA was assessed by immunofluorescence and western blotting in A549 cells. Tubulin was used as a loading control.

SUPPLEMENTAL EXPERIMENTAL PROCEDURES

Cell Culture and irradiation

A549, HeLa and U2OS cells were cultured in DMEM (Gibco) with 10% FCS, L-glutamine, penicillin and streptomycin (Gibco) at 37°C in a humidified 95% air and 5% CO^2 atmosphere. Cells were irradiated by exposure to a ^{137}Cs source. U2OS reporter cells were cultured in DMEM (Gibco) with 10% Tet-System approved FBS (Clontech), L-glutamine, penicillin and streptomycin (Gibco), 200mg/ml G418 and 100mg/ml Hygromycin B, at 37°C in a humidified 95% air and 5% CO2 atmosphere.

Premature chromosome condensation (PCC) breakage analysis.

PCC analysis was carried out as described previously (Deckbar et al., 2007). Briefly, irradiated (2 Gy) HeLa cells were treated at the indicated time points with 50 ng/ml Calyculin-A for 0.5h to induce PCC in G2-phase cells. Cells were then harvested, fixed, and processed for chromosome break analysis. Chromosome breaks were scored per 100 chromosomes counted. All results represent the mean +/- SD of three experiments.

Laser microirradiation.

Exponentially growing human U2OS cells were plated onto 35mm glass-bottom dishes (MatTek) and transfected with the pEGFP-BAF180, GFP-XRCC4 or GFP-BRG1 constructs using NanoJuice according to the manufacturers protocol. The cells were allowed to express the construct for 24h and were then incubated with 10 µg/ml Hoechst 3458 for 1 h at 37 °C before irradiation. The microscope system used was an Intelligent Imaging Innovations spinning disk confocal with a Yokogawa CSU-X1 on an Olympus IX-71. GFP positive cells were irradiated with a 100mW, 405nm ultraviolet laser set at 20% power and channelled through a 60x objective. The UV laser was focused to an area of approximately 12 µm*0.1µm through the cell nuclei, and images were captured at 10s intervals following laser damage for a total time of 180s. Images generated were acquired on a Photometrics Evolve 512x512 EMCCD using Slidebook 5.5 software. Where indicated, 10 µMATMi (Tocris Bioscience) was added 1h prior to irradiation. In protein recruitment experiments, signal intensity was quantified along the laser path, using Slidebook 5.5 software, in a minimum of 20 cells and error bars represent the SD between three independent experiments.

Small interfering RNA (siRNA) knockdown conditions

siRNA-mediated knockdown was achieved using HiPerFect Transfection Reagent (Qiagen, Hilden, Germany) following the manufacturer’s instructions.siRNA duplexes were

transfected into 2 x 10^5 of logarithmically growing cells per condition. Cells were harvested 24h later, re-transfected with siRNA and then seeded and grown for 48 hours. The BAF180 siRNA oligonucleotide is 5′-GAGAAATCTTGAGACAGCCAAGAAA-3′ (Invitrogen Stealth RNAi). The BRG1 siRNA is a pool of 3 target specific siRNAs from Santa Cruz. For BRG1 complementation experiments the siRNA oligonucleotide used is 5'-CGCGGCACATCATTGAGAATGCCAA-3'. The RNF8 siRNA was described previously (Mailand et al., 2007). The BMI1 and EZH2 siRNA oligonucleotides are from the Dharmacon smartpool.

Antibodies

The primary antibodies used were: γH2AX (Upstate Technology) at 1:800 for IF, anti-phospho Ser2 RNAPII (Abcam) at 1:500 for IF, Anti-monoubiquityl-Histone H2A (Lys119) (Upstate Technology) at 1:500 for (IF), FK2 (Enzo) at 1:400 for IF, BAF180 (Bethyl) at 1:2000 for WB, α-Tubulin (Abcam) at 1:5000 for WB, KAP1 (Abcam) 1:1000 for WB, EZH2 (New England Bioloabs) 1:400 for IR and 1:1000 for WB, BMI1 (Cambridge Bioscience) 1:400 for IF and 1:1000 for WB.

The secondary antibodies used were: FITC (Sigma Aldrich) at 1:200 for IF, CY3 (Sigma Aldrich) at 1:200 for IF, Alexa647 (Invitrogen) at 1:400 for IF, Polyclonal Goat Anti-Rabbit Immunoglobulins/HRP at 1:2000 for WB, Polyclonal Rabbit Anti-Mouse Immunoglobulins/HRP at 1:2000 for WB.

Immunofluorescence

Cells plated on glass slides were fixed for 10 min with fixative (3% (w/v) PFA, 2% (w/v) sucrose, 1X PBS) and permeabilized for 1 min with 0.2 % Triton X-100 in PBS. When staining for H2AK119ub, pre-extraction was performed by treatment with 0.2% Triton X-100 in PBS for 0.5-1 min prior to PFA fixation. Cells were rinsed with PBS and incubated with primary antibody diluted in PBS + 2% (w/v) BSA for 1 h at room temperature (RT). Cells were washed three times, incubated with secondary antibody (diluted in PBS + 2% (w/v) BSA) for 30 min at RT in the dark, incubated with 4′,6-diamidino-2-phenylindole (DAPI) where indicated for 10 min, and washed three times with PBS. Slides were mounted using Vectashield and visualised/analysed using a Nikon-e400 microscope and imaged using an Applied Precision® Delta Vision® RT Olympus IX70 deconvolution microscope and softWoRx® Suite software. For γH2AX foci quantification, a minimum of 30 cells was scored blindly and error bars represent the SD between three independent experiments. In complementation experiments, IRIF were only scored in GFP expressing cells.

H2AK119ub and FK2 immunofluorescence signal quantification was carried out using ImageJ. The "Spot Analyser" plugin and the associated script are freely available at http://www.sussex.ac.uk/gdsc/intranet/microscopy/imagej/utility. Here, cell nuclei were detected using Otsu's thresholding method (Otsu, 1979). The plugin smooths the image using a Gaussian blur to aid spot detection on noisy data. In this case, panuclear YFP signal was used to detect cell nuclei in the U2OS cells containing the transcriptional reporter system. Next, the plugin was used to detect the reporter site (mCherry expression) within the nucleus by searching for intensity maxima above the background level. The background is set using the mean intensity within the nuclei.The maxima are enlarged using a watershed algorithm of steepest descent. The final maxima region is then pruned back to a fraction of its height above the background. Subsequently, fluorescence intensity measurements inside and outside the maxima were obtained for H2AK119ub and FK2, and expressed as a ratio. In summary, the following parameters were used: Threshold method- Otsu, Blur- 3, Minimum particle size- 2000, Number of peaks- 1, Fraction- 0.9. A minimum of 10 cells were analysed per condition from three independent experiments. The data are normalised to the measurements in control cells. Error bars represent SD.

Quantification of transcription status in U2OS transcription reporter cells.

Transcription status was monitored in U2OS reporter cells following treatment with the indicated siRNAs and following transient expression of the indicated constructs. U2OS reporter cells were plated on glass coverslips and transfected with 1μg mCherryFokI using *NanoJuice*® transfection reagent. 24h later, 1μg/ml doxycycline was added for three hours. Where indicated, 10 μM ATMi (Tocris Bioscience) was added to the cells 60 minutes prior to the addition of doxycycline. Where indicated, 100 μM DRB for was added for 3 hr prior to the addition of doxycycline. Following doxycycline treatment, cells were fixed and mounted as described above. Transcription status was monitored using a Nikon-e400 microscope by quantifying YFP positive cells at the transcription reporter site, located by mCherry signal. A minimum of 100 cells were analysed blindly and error bars represent the SD between three independent experiments.

Plasmids and Constructs

The mCherryFokI construct was described previously (Shanbhag et al., 2010). BAF180 complete cDNA (clone MGC:156155, IMAGE:40082629) was purchased from Source BioScience and cloned into *Hind*II/*Kpn*I sites of pEGFP-C3 (Clontech). This clone corresponds to isoform 8 and contains a N122S substitution relative to the published sequence. To generate siRNA-resistant BAF180 expression constructs, T285C, G291A and C297T silent point mutations were introduced into the BAF180 cDNA of pEGFP-

BAF180 by site directed mutagenesis (QuickChange, Agilent), using the oligonucleotides 5′- GTGGGGACAGAATGGAGAAACCTTGAAACAGCTAAGAAAGCAGAATATGAAG-3′ (forward) and 5'-CTTCATATTCTGCTTTCTTAGCTGTTTCAAGG TTTCTCCATTCTGTCCCCAC-3' (reverse) to create pEGFP-BAF180r. The pEGFP-BAF180r^{S963A}, and pEGFP-BAF180r^{S963E} constructs were generated by site directed mutagenesis (QuickChange, Agilent). The pEGFP-BAF180r^{T232P}and pEGFP-BAF180r^{M538I} constructs were described previously (Brownlee et al., 2014).

GFP-tagged, human BRG1 cDNA ORF clone (NM_001128849.1) was purchased in the pCMV6-AC-His vector from Origene. To generate siRNA-resistant BRG1 expression constructs, G2186A, C2192A, G2198A and C2204A silent point mutations were introduced into the cDNA of GFP-BRG1 by site directed mutagenesis (QuikChange Lightning Multi Site-Directed Mutagenesis, Agilent) using the oligonucleotide 5'-CTGAGGTGGACGCGCGACACATAATTGAAAATGCGAAGCAAGATGTCGATG-3'. The K798R mutation in the siRNA resistant GFP-BRG1 expression construct was generated using the oligonucleotide 5'-ATGGGCCTGGGGAGGACCATCCAGACC-3'.

SUPPLEMENTAL REFERENCES

Brownlee, P.M., Chambers, A.L., Cloney, R., Bianchi, A., and Downs, J.A. (2014). BAF180 Promotes Cohesion and Prevents Genome Instability and Aneuploidy. Cell reports *6*, 973-981.

Deckbar, D., Birraux, J., Krempler, A., Tchouandong, L., Beucher, A., Walker, S., Stiff, T., Jeggo, P., and Lobrich, M. (2007). Chromosome breakage after G2 checkpoint release. J Cell Biol *176*, 749-755.

Mailand, N., Bekker-Jensen, S., Faustrup, H., Melander, F., Bartek, J., Lukas, C., and Lukas, J. (2007). RNF8 Ubiquitylates Histones at DNA Double-Strand Breaks and Promotes Assembly of Repair Proteins. Cell *131*, 887-900.

Otsu, (1979). A Threshold Selection Method from Gray-Level Histograms.IEEE Transactions *9*, 62-66.

Shanbhag, N.M., Rafalska-Metcalf, I.U., Balane-Bolivar, C., Janicki, S.M., and Greenberg, R.A. (2010). ATM-dependent chromatin changes silence transcription in cis to DNA double-strand breaks. Cell *141*, 970-981.
