# Supplementary material for: Requirement for PBAF in Transcriptional Repression and Repair at DNA Breaks in Actively Transcribed Regions of Chromatin
Source: Mol Cell. 2014 Sep 4;55(5):723–32. doi: 10.1016/j.molcel.2014.06.028 (PMC4157577; doi:10.1016/j.molcel.2014.06.028)
Supplement: Document S2. Article plus Supplemental Information [file mmc2.pdf]

Requirement for PBAF in Transcriptional Repression and Repair at DNA Breaks in Actively Transcribed Regions of Chromatin

Andreas Kakarougkas,[1] Amani Ismail,[1] Anna L. Chambers,[1] Enriqueta Riballo,[1] Alex D. Herbert,[1] Julia Künzel,[2] Markus Löbrich,[2] Penny A. Jeggo,[1,*] and Jessica A. Downs[1,*]
[1]MRC Genome Damage and Stability Centre, University of Sussex, Falmer, Brighton BN1 9RQ, UK
[2]Radiation Biology and DNA Repair, Darmstadt University of Technology, 64287 Darmstadt, Germany
*Correspondence: p.a.jeggo@sussex.ac.uk (P.A.J.), j.a.downs@sussex.ac.uk (J.A.D.)
http://dx.doi.org/10.1016/j.molcel.2014.06.028
This is an open access article under the CC BY license (http://creativecommons.org/licenses/by/3.0/).

SUMMARY

Actively transcribed regions of the genome are vulnerable to genomic instability. Recently, it was discovered that transcription is repressed in response to neighboring DNA double-strand breaks (DSBs). It is not known whether a failure to silence transcription flanking DSBs has any impact on DNA repair efficiency or whether chromatin remodelers contribute to the process. Here, we show that the PBAF remodeling complex is important for DSB-induced transcriptional silencing and promotes repair of a subset of DNA DSBs at early time points, which can be rescued by inhibiting transcription globally. An ATM phosphorylation site on BAF180, a PBAF subunit, is required for both processes. Furthermore, we find that subunits of the PRC1 and PRC2 polycomb group complexes are similarly required for DSB-induced silencing and promoting repair. Cancer-associated BAF180 mutants are unable to restore these functions, suggesting PBAF's role in repressing transcription near DSBs may contribute to its tumor suppressor activity.

INTRODUCTION

Transcription needs to be carefully regulated to interface with DNA metabolic processes that serve to maintain genome stability. Recently, it was found that, in response to a euchromatic DNA double-strand break (DSB), a signal is sent in *cis* to repress transcription in flanking chromatin (Shanbhag et al., 2010). This is dependent on the ATM kinase and involves H2A monoubiquitination at K119 (H2AK119ub; Shanbhag et al., 2010). While it is currently not known what advantage this gives to the cell, failure to silence transcription in chromatin flanking a DSB might be predicted to affect DSB repair.

While H2AK119ub is genetically dependent on the RNF8 and RNF168 ubiquitin ligases (Shanbhag et al., 2010), these cannot catalyze this modification in vitro (Mattiroli et al., 2012). Instead, there is evidence that H2AK119ub is catalyzed by polycomb group (PcG) proteins (Cao et al., 2005; Mattiroli et al., 2012). There are two main PcG complexes: polycomb repressive complex 1 (PRC1) and 2 (PRC2). The EZH2 methyltransferase is found within PRC2 and is known to promote H3 K27me3. At PcG-regulated promoters, this modification facilitates PRC1 recruitment, which represses transcription by promoting H2A monoubiquitination at K119, and this is dependent on the BMI1 subunit of PRC1 (Cao et al., 2005).

Both PRC2 and PRC1 have been implicated in DNA DSB responses (Vissers et al., 2012). Subunits present in both complexes are recruited to sites of DNA damage, and loss of either PRC2 or PRC1 results in decreased survival following irradiation (IR) and modest DNA repair defects (Vissers et al., 2012). Given their known role in gene regulation and promoting H2AK119ub, these complexes are excellent candidates for mediating DSB-induced transcriptional silencing.

The PBAF chromatin remodeling complex (also termed SWI/SNF-B) is one of two SWI/SNF complexes found in mammalian cells, which have considerable overlap in subunit composition. While there is evidence that loss or depletion of subunits common to both complexes causes defects in DNA DSB repair (Chambers and Downs, 2012; Papamichos-Chronakis and Peterson, 2013), it is not clear whether or how PBAF contributes to this outcome. The catalytic subunit, BRG1, is found in both complexes; however, three subunits—BAF200, BAF180, and BRD7—are specific to the PBAF complex. The gene encoding BAF180 is frequently mutated in human tumors (Shain and Pollack, 2013), which may reflect a role in maintaining genome stability, and we and others have demonstrated that the yeast homologs of BAF180 contribute to the repair of DSBs (Chambers and Downs, 2012).

We set out to investigate whether any chromatin remodeling complexes might be required for DSB-induced transcription arrest and found that PBAF functions in the ATM pathway silencing transcription in *cis* to DSBs. PBAF does not influence ubiquitin chain formation at DSBs or the canonical downstream DNA damage signaling response but specifically promotes H2AK119ub. Furthermore, we uncovered a subtle role for PBAF in the efficiency of repair of DSBs by nonhomologous end joining (NHEJ) that can be rescued by globally inhibiting transcription, demonstrating that there is a consequence to the

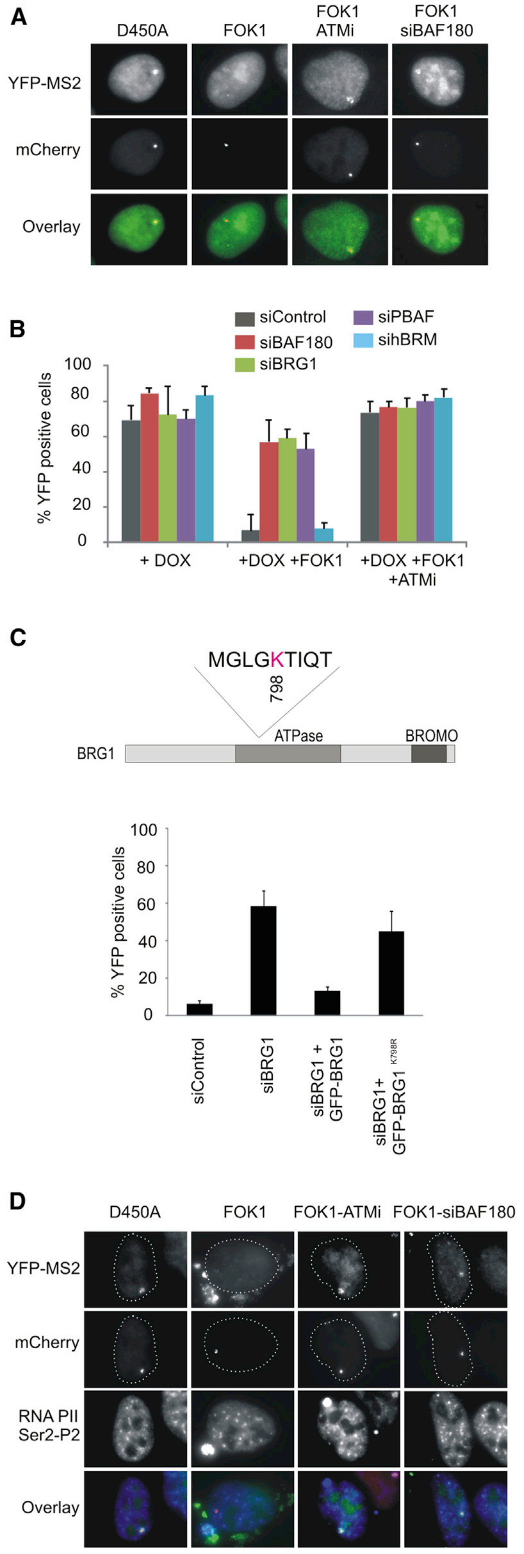

Figure 1. PBAF Is Required for Transcriptional Silencing Induced by DNA DSBs

(A) Representative immunofluorescence images of U2OS reporter cells expressing mCherry-tagged WT or nuclease-deficient (D450A) FokI (Shanbhag et al., 2010). On addition of doxycycline, YFP signal accumulation at the reporter site is a result of YFP-MS2 protein binding to MS2 stem loops and is indicative of nascent transcript formation. Following transfection of WT FokI, but not the nuclease-deficient Fok1 (D450A), DSBs are induced at the reporter site and transcriptional silencing occurs, leading to loss of YFP signal. Treatment with ATMi or siBAF180 leads to persistent YFP signal formation at the reporter site in WT FokI-expressing cells indicating deficient transcriptional silencing in response to DNA DSBs.

(B) Quantification of doxycycline (DOX)-induced transcription in U2OS reporter cells treated with the indicated siRNA. Transcriptional silencing was monitored after addition of doxycycline by quantification of YFP-positive cells expressing WT FokI endonuclease (FOK1) in the presence and absence of ATMi. Data are represented as mean ± SD.

(C) Top: cartoon showing the location of K798 in the catalytic subunit of PBAF (BRG1). Bottom: siRNA-resistant WT or K798R mutant BRG1 expression constructs were introduced into siBRG1 cells and assayed for transcriptional silencing in cells expressing WT FokI endonuclease by quantification of YFP-positive cells. Data are represented as mean ± SD. BROMO, bromodomain.

(D) Actively elongating RNA polymerase II (RNAPII) at the reporter site was monitored using an antibody against phosphorylated Ser2 of the C-terminal domain of the RNAPII large subunit.

See also Figure S1.

cell if transcription silencing in *cis* to DSBs fails. In addition, we tested the potential role of the PcG complexes in this pathway and found that the PcG complexes PRC2 and PRC1 are important for mediating both DNA damage-induced transcriptional silencing and rapid repair of DSBs. Notably, neither phenotype can be rescued by BAF180 constructs bearing point mutations found in cancer samples, highlighting the potential significance of this pathway to the role of BAF180 in preventing tumorigenesis.

RESULTS

PBAF Silences Transcription Flanking DNA Breaks

A recent study identified a role for ATM in silencing transcription in *cis* to DSBs by promoting histone H2A monoubiquitylation on lysine 119 (Shanbhag et al., 2010). We predicted that such transcriptional silencing might require chromatin remodeling factors. To test this prediction, we made use of the reporter cells developed by the Greenberg lab, in which DSBs can be induced at defined regions upstream of an inducible reporter gene (Shanbhag et al., 2010). Lac operator repeats are used to target either wild-type (WT) or nuclease-deficient mutant FokI endonuclease to the reporter locus, where an mCherry tag allows visualization of the locus (Figure 1A). Addition of doxycycline drives the expression of the reporter gene, a process that is visualized by the production of nascent transcript (yellow fluorescent protein [YFP]; Figure 1A), and this is unaffected by expression of the nuclease-deficient mutant (FokI-D540A). In cells expressing WT FokI, however, no nascent transcript is observed on addition of doxycycline since the DSBs upstream of the promoter lead to transcriptional silencing (Figure 1A). Consistent with previous findings, this process is ATM dependent, and transcription persists in the presence of DSBs in ATM inhibitor (ATMi)-treated cells (Figures 1A and 1B).

Strikingly, we observed that depletion of BAF180 and/or BRG1 resulted in a failure to silence transcription in FokI-expressing cells, albeit to a slightly lesser extent than ATMi-treated cells

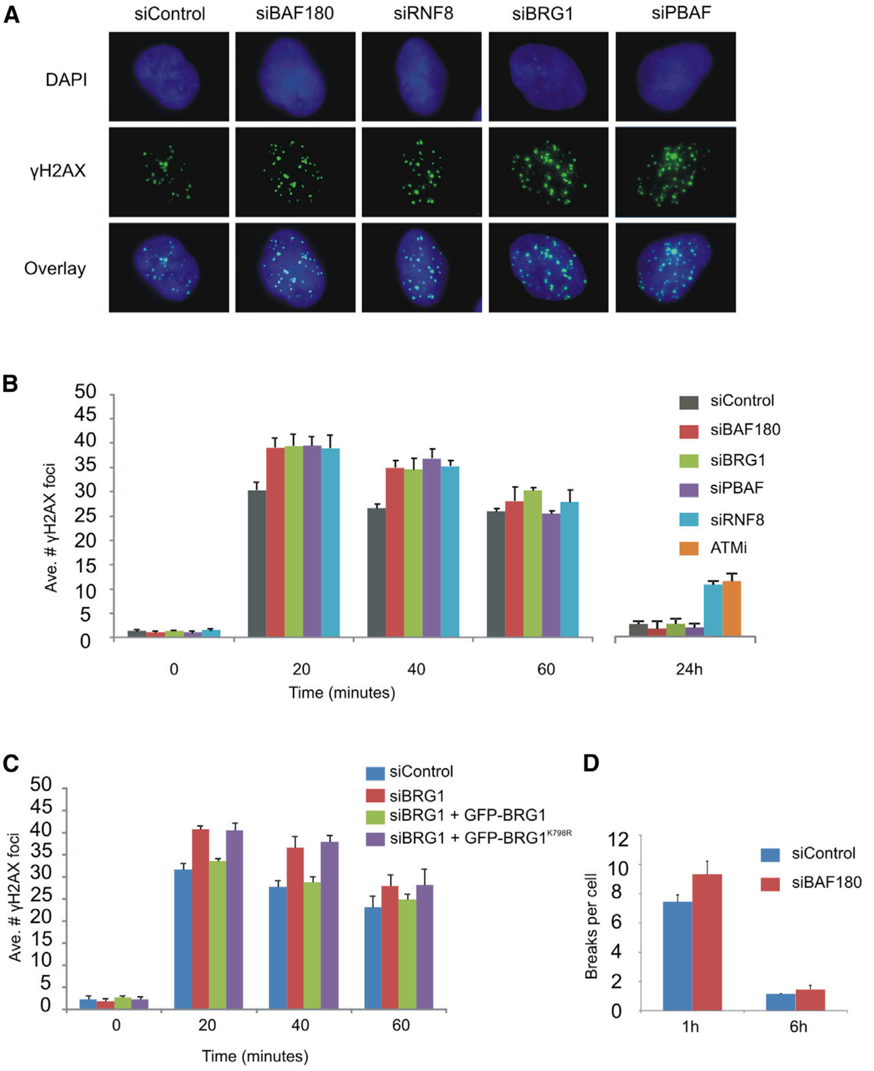

Figure 2. PBAF Contributes to NHEJ at Early Time Points following DNA Damage

(A) Representative immunofluorescence images of A549 cells treated with the indicated siRNA, 40 min following exposure to 1.5 Gy IR.

(B) Quantification of γH2AX foci clearance following exposure to 1.5 Gy IR in A549 cells treated with the indicated siRNA or ATMi. Data are represented as mean ± SD. h, hours; Ave., average.

(C) siRNA-resistant WT or K798R mutant BRG1 expression constructs were introduced into siBRG1 cells and assayed for γH2AX foci clearance following exposure to 1.5 Gy IR. Data are represented as mean ± SD.

(D) Chromosome breakage analysis in siControl- and siBAF180-treated 82-6 hTert fibroblasts at early and late times following 7 Gy IR. Data are represented as mean ± SE.

See also Figures S2 and S3.

(Figures 1A and 1B). This is specifically dependent on PBAF, since we find that depletion of the catalytic subunit of the related BAF complex (hBRM) has no effect on this activity (Figure 1B; Figure S1A available online). To determine whether the catalytic activity of PBAF is required, we created a small interfering RNA (siRNA)-resistant BRG1 expression construct and introduced a mutation (K798R) that has previously been shown to abrogate BRG1 activity (Khavari et al., 1993) (Figure 1C). Consistent with previous reports, we find that both the WT and K798R mutant constructs are stably expressed in vivo (Figure S1B), yet only reintroduction of the WT, but not the K798R, mutant BRG1 expression construct restored DNA damage-dependent transcriptional silencing in BRG1-depleted cells (Figure 1C), indicating that the remodeling activity of PBAF is required for this activity. Finally, hyperaccumulation of the active form of RNA polymerase II was observed at the locus in ATMi-treated and BAF18-depleted cells, despite DSB induction by FokI expression (Figure 1D). Taken together, these findings suggest that PBAF functions in the ATM-dependent pathway leading to transcriptional silencing in *cis* to DSBs.

PBAF Contributes to NHEJ at Early Time Points following DNA Damage

We predicted that any impact of a failure to inhibit transcription at DSBs on global levels of DSB repair would be subtle and therefore chose γH2AX foci clearance as a sensitive readout of DNA DSB repair. We examined repair in G0/G1 cells to avoid any impact of roles of PBAF during replication. Notably, we observed a modest but reproducible increase in γH2AX foci at early, but not late, time points post-IR in cells depleted for the BAF180 subunit and/or the BRG1 catalytic subunit of the PBAF complex (Figures 2A and 2B). Similar to what we found when the repression of transcription flanking a DSB was investigated, we found that the catalytic mutant of BRG1 is unable to rescue this phenotype (Figure 2C), suggesting that chromatin remodeling is required for the ability of PBAF to promote efficient repair at early time points following IR.

These data suggest that, in the absence of PBAF, there is a delay in the repair of a subset of DSBs at early time points. Consistent with this possibility, we find that both BAF180 and BRG1 rapidly localize to laser-induced tracks of DNA damage (Figures S2A and S2B). The pattern of localization, in contrast to the tracks formed by γH2AX or XRCC4 (Figure S2C), appears to show accumulation of PBAF in chromatin flanking the damaged area rather than directly within it, perhaps suggesting movement away from the damage in *cis* to chromatin flanking the break.

In addition to using γH2AX as a readout of DSB repair, we directly monitored chromosome breakage following IR in 82-6 human telomerase reverse transcriptase (hTert) fibroblast cells promoted to undergo condensation by fusion with mitotic HeLa cells. Consistent with our results with γH2AX foci, we observed a small increase in chromosomal breakage in

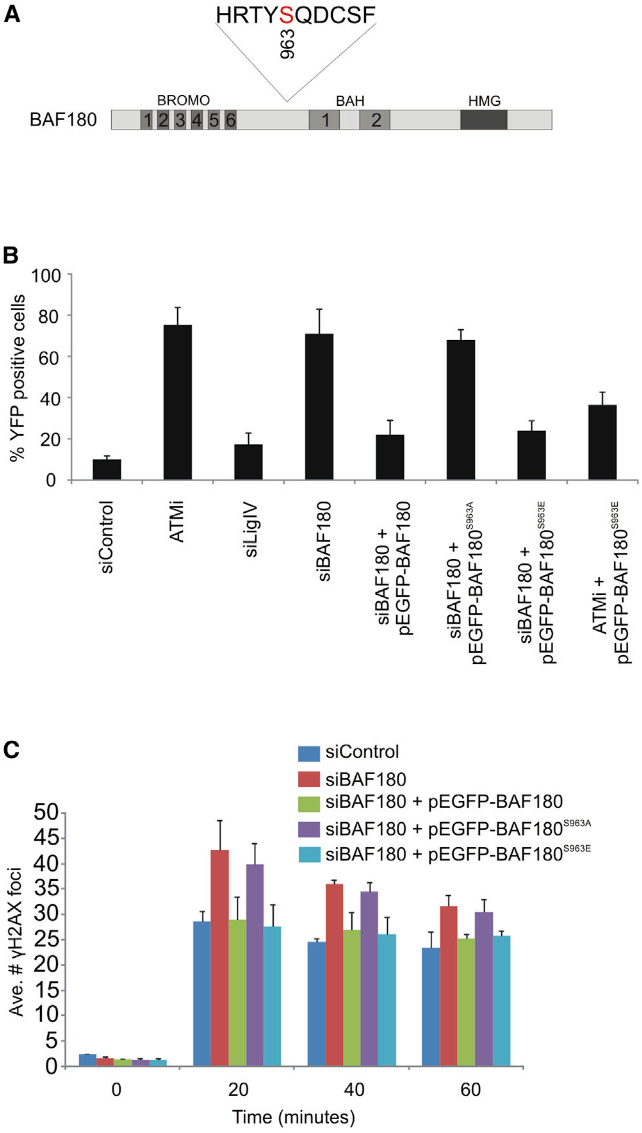

Figure 3. Phosphorylation of BAF180 by ATM Is Required for Transcriptional Repression and Early DNA Repair Activity

(A) Cartoon of human BAF180 highlighting the domain organization and the sequence surrounding serine 963 (Ser 948 in isoform 1), which is phosphorylated by ATM in response to DNA damage.

(B) Quantification of YFP-positive cells as a measure of DSB-induced transcriptional repression in U2OS reporter cells treated with the indicated siRNA and complemented with BAF180 expression constructs as indicated. Data are represented as mean ± SD.

(C) Quantification of γH2AX foci clearance following exposure to 1.5 Gy IR in U2OS cells treated with the indicated siRNA and complemented with BAF180 expression constructs as indicated. Data are represented as mean ± SD. Ave., average.

See also Figures S1 and S4.

BAF180-depleted cells compared to WT controls at 1 hr post-IR (Figure 2D), suggesting that PBAF is required for a process of DSB repair that occurs rapidly after IR. Because these effects are subtle, we used an additional method in another cell line to directly monitor breaks. In this approach, we treated irradiated HeLa cells with calyculin, which causes premature chromosome condensation of G2 cells. Here, again we observed enhanced chromosome breaks after BAF180 depletion at early, but not late, time points following IR (Figure S3A).

While we are unable to examine the requirement for ATM for DSB repair at early times since γH2AX foci form with delayed kinetics in ATMi or siRNA ATM-treated cells (Stiff et al., 2004), depletion of RNF8 conferred a similar increase in γH2AX foci at early time points (Figure 2B). Consistent with previous studies, addition of an ATMi or depletion of RNF8 causes defective γH2AX foci clearance at late times, due to failure to repair DSBs located at heterochromatic regions (Noon et al., 2010) (Figure 2B). PBAF does not appear to function in this aspect of the DNA damage response (Figure 2B). Together, these results indicate that the ATM signaling pathway that has been previously shown to function at slowly repaired DSBs does not require PBAF, and furthermore, that PBAF is required for a rapid repair process that is also dependent on RNF8.

ATM and RNF8 facilitate the repair of DNA DSBs in both the G1 and G2 phases of the cell cycle, regardless of which repair pathway is used (Goodarzi and Jeggo, 2013). We wanted to investigate whether the early repair process requiring PBAF identified here involves NHEJ proteins or a distinct repair process. We monitored γH2AX foci clearance following BAF180 depletion at early and late times post-IR and compared it to the phenotype of cells depleted for DNA ligase IV (LigIV), a core NHEJ component (Figures S3B and S3C). LigIV-depleted cells showed a severe repair defect, particularly by 24 hr post-IR, consistent with its central role in NHEJ. In addition, LigIV-depleted cells showed a small but significant repair defect at early time points, demonstrating that in WT cells, some DSBs have undergone repair by NHEJ at these early time points. This defect is similar to that observed in BAF180-depleted cells (Figures S3B and S3C). It is important to note that concurrent depletion of BAF180 and LigIV did not lead to an additive repair defect (Figures S3B and S3C), strongly suggesting that BAF180/PBAF facilitates the rapid repair of a subset of DSBs by NHEJ.

Phosphorylation of BAF180 by ATM Is Required for Transcriptional Repression and Early DNA Repair Activity

Next, we aimed to gain functional insight into the relationship between ATM and BAF180. A large proteomics study identified a damage regulated SQ/TQ site on serine 948 of BAF180 (Matsuoka et al., 2007) (Figure 3A), which corresponds to S963 in our BAF180 expression construct (isoform 8). To test the functional significance of this site, we generated phosphomutant (S963A) and phosphomimic (S963E) versions of siRNA-resistant BAF180 constructs (Figures S1C and S1D) and investigated whether these could complement BAF180-depleted cells. We first established that reconstitution of BAF180-depleted cells with siRNA-resistant WT BAF180 restores transcriptional silencing, thus eliminating the possibility of off-target effects of siRNA (Figure 3B). Significantly, although expression of the phosphomimic construct also rescued the silencing defect of siBAF180 cells, the phosphomutant construct did not (Figure 3B), suggesting that phosphorylation of BAF180 by ATM is critical for its function in this assay. Strikingly, we found that expression of

the phosphomimic BAF180 construct was able to partially rescue the silencing defect observed in cells treated with the ATM inhibitor (Figure 3B).

We further investigated the effect of these mutations on early DSB repair. Reconstitution of siRNA BAF180 cells with WT and phosphomimic BAF180 led to repair kinetics that were indistinguishable from control cells, whereas expression of the phosphomutant construct did not rescue the repair defect of BAF180-depleted cells (Figure 3C). We investigated the possibility that ATM-dependent phosphorylation of PBAF promotes recruitment to DSBs, but we found that BAF180 localization to damage is unaffected by ATM inhibition (Figure S4). Nevertheless, these findings strengthen the notion of functional interplay between ATM and BAF180 in transcriptional silencing in *cis* to DSBs, which is a prerequisite for efficient DSB repair at early times post-IR.

The Role for BAF180 in Early DSB Repair Requires Active Transcription

We hypothesized that the inability to silence transcription of genes flanking DSBs may be responsible for the delayed repair of the subset of DSBs observed in BAF180- and RNF8-depleted cells at early times post-IR. To test this, we treated cells with the transcriptional inhibitor 5,6-Dichlorobenzimidazole 1-b-D-ribofuranoside (DRB), which efficiently inhibits transcription in doxycycline-treated reporter cells (Figure 4A). DRB treatment had no effect on the repair kinetics of siControl cells when repair was monitored either by γH2AX foci clearance or by chromosome break analysis (Figures 4B, 4C, and S3A). In contrast, the early repair delay in siBAF180 cells was rescued by DRB treatment (Figures 4B, 4C, and S3A), indicating that, in the absence of ongoing transcription, PBAF is dispensable for efficient DSB repair following IR. This demonstrates that the lack of transcriptional repression of genes flanking DNA DSBs has a consequence; it impedes DSB repair.

PBAF Is Specifically Required for H2A K119 Ubiquitination at DSBs

It has recently been shown that small RNAs are produced at chromatin flanking DSBs in a manner dependent on DICER and DROSHA (Francia et al., 2012; Wei et al., 2012), and these are required for ATM signaling (Francia et al., 2012). One mechanism by which PBAF may function to promote DSB repair is by facilitating the transcription of these RNA species. DICER and DROSHA are upstream of ATM in the DNA damage signaling response, and ATM phosphorylation is defective in the absence of small RNA formation (Francia et al., 2012). We therefore investigated the effect of BAF180 depletion on ATM activation and found that, in contrast to DICER depletion, loss of PBAF has no detectable effect on phospho-ATM (Figure S5), suggesting that PBAF is not required for damage-dependent RNA formation.

To gain insight into how PBAF might affect the process of transcriptional silencing, we examined whether, like ATM, PBAF promotes monoubiquitination of H2A K119 at transcriptionally active DSBs. Using an antibody specific for this histone modification, we found an enrichment of H2AK119ub at the locus site in cells expressing WT FokI, and this coincided with loss of active transcription as expected (Figure 4D). Significantly, BAF180 depletion or ATMi treatment led to persistent transcription in FokI-expressing cells, which coincided with a significant reduction in H2AK119ub enrichment at the reporter locus (Figures 4D and 4E).

H2A is also ubiquitinated on K13–K15 (Mattiroli et al., 2012). It is first monoubiquitinated by RNF168, and then K63-linked ubiquitin chains are extended from this site (Mattiroli et al., 2012). The monoubiquitinated K15 form of H2A is recognized by 53BP1, which also forms foci following IR treatment (Fradet-Turcotte et al., 2013). In BAF180-depleted cells, we found no defect in 53BP1 foci formation, suggesting that H2AK15ub and subsequent binding by 53BP1 are unaffected (Figure 4F). We also monitored the accumulation of ubiquitinated proteins at DSBs using the FK2 antibody and found that FK2 foci are unaffected by depletion of BAF180 (Figures 4F and 4G). These data suggest that H2A K15 ubiquitination and downstream signaling events are unaffected by PBAF.

Our results show that BAF180 is specifically required for events leading to DSB-induced transcriptional repression and that this impacts on the ability of cells to repair a subset of DSBs with fast kinetics, raising the possibility that PBAF is specifically recruited to those DSBs flanking actively transcribed genes. Both BAF180 (Figure S1) and H2AK119ub (Ginjala et al., 2011) localize to laser tracks, which may reflect global recruitment to all DSBs. To test this further, we investigated H2AK119ub foci formation following IR. We found that H2AK119ub foci form and colocalize with 53BP1 foci following IR treatment (Figure 4F), consistent with the possibility that H2AK119ub is not limited to DSBs flanking actively transcribed genes. In addition, we found that global inhibition of transcription using the inhibitor DRB did not abolish H2AK119ub foci formation (data not shown). Notably, similar to what was found when ATM is inhibited (Shanbhag et al., 2010), H2AK119ub foci are reduced in BAF180-depleted cells (Figures 4F and 4G). These data suggest that, although BAF180 only functions to promote the rapid repair of a subset of breaks, it is required for efficient H2AK119ub foci formation at most or all DSBs.

The PcG Proteins BMI1 and EZH2 Are Required for Both DNA Damage-Induced Transcriptional Silencing and Efficient DSB Repair at Early Time Points

As described earlier, the PcG complexes PRC1 and PRC2 have been implicated in DNA DSB responses. The PRC1 complex, which contains the BMI1 subunit, ubiquitinates H2A K119 both at promoters and at sites of damage, and depletion of BMI1 results in a global loss of H2AK119ub foci in irradiated cells (Ginjala et al., 2011) (Figure 5A), which is similar to what we find when BAF180 is depleted (Figure 3F). We therefore depleted BMI1 (Figure S6) and monitored the ability of cells to repress transcription in response to a Fok1-induced DSB. In doing so, we found that damage-induced transcriptional silencing is impaired when BMI1 is depleted (Figure 5B), implicating the PRC1 complex in this pathway.

At promoters, PRC2 promotes H3 K27me3, which is recognized by PRC1. It has been shown that PRC2, like PRC1, contributes to the DNA DSB response, suggesting that it may function similarly to recruit PRC1 to DSBs (Campbell et al., 2013; Chou et al., 2010). However, there is no consensus on whether H3

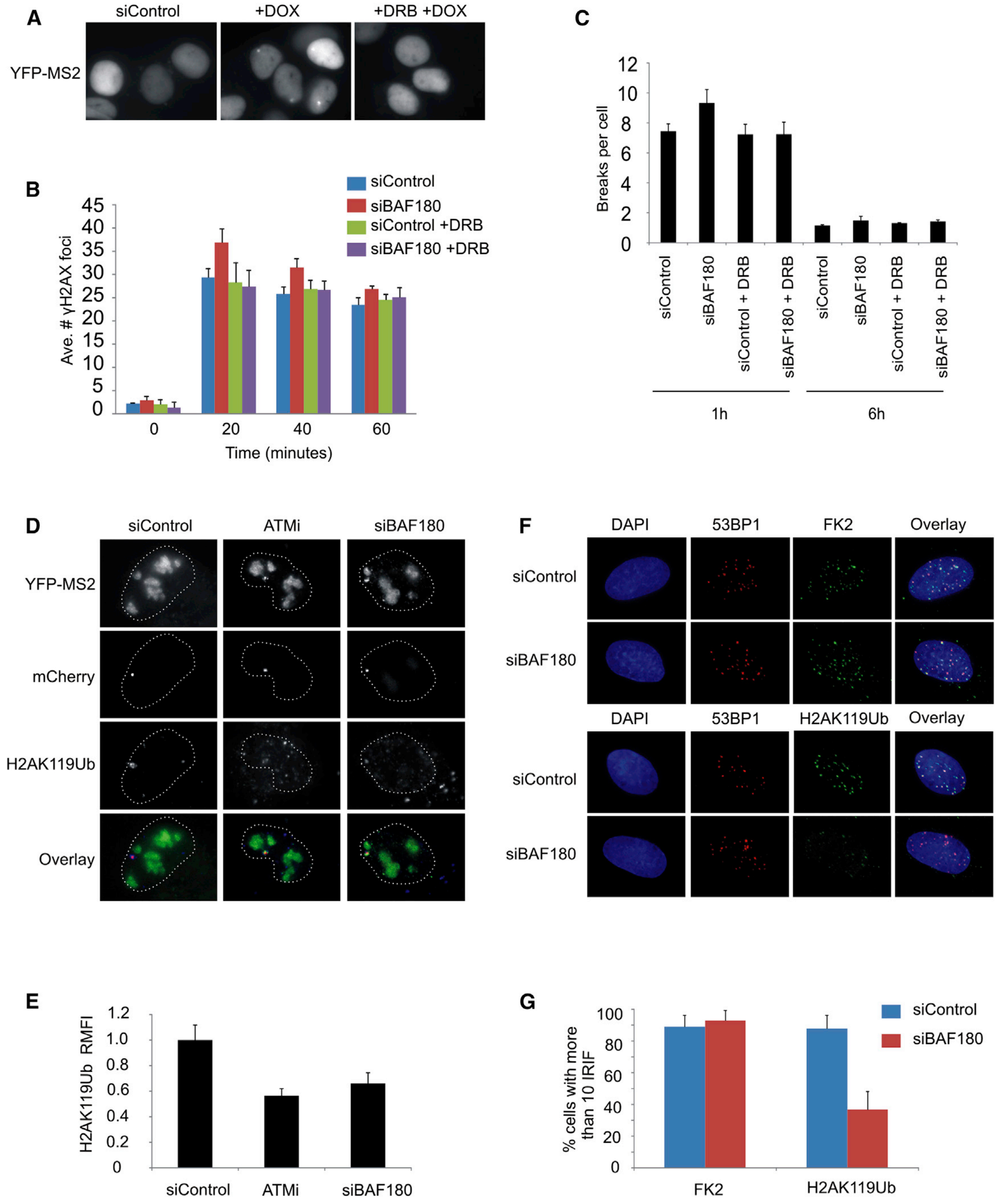

Figure 4. PBAF Is Required for Efficient Repair Only when There Is Ongoing Transcription and Promotes H2A K119 Ubiquitination at DSBs

(A) Treatment of U2OS reporter cells with DRB efficiently inhibits transcription, as demonstrated by the loss of YFP signal at the reporter site of doxycycline-treated cells.

(B) Quantification of γH2AX foci clearance following exposure to 1.5 Gy IR in control and siBAF180 A549 cells with and without DRB treatment prior to IR. Data are represented as mean ± SD. Ave., average.

(C) Chromosome breakage analysis in siControl- and siBAF180-treated 82-6 hTert fibroblasts at early and late times following 7 Gy IR with the addition of DRB (data without DRB are as in Figure 2C). Data are represented as mean ± SE. h, hours.

(D) Representative immunofluorescence images of YFP, mCherry, and H2AK119ub following DSB induction in control, ATM inhibitor-treated, or siBAF180 cells.

(E) Relative mean fluorescence intensity (RMFI) of H2AK119Ub signal at the reporter site of WT FokI expressing cells treated with ATMi or siBAF180, relative to control cells. Data are represented as mean ± SD.

(legend continued on next page)

K27me3 is involved in this (Campbell et al., 2013; Chou et al., 2010), and it has not been investigated whether PRC2 is required for ionizing radiation-induced H2AK119ub foci. We depleted the EZH2 subunit of PRC2 (Figure S6) and found that, similar to BAF180 and BMI1 depletion, there are fewer H2AK119ub foci in irradiated cells (Figure 5A), suggesting that PRC2 promotes PRC1 activity at sites of DNA damage.

It is important to note that we find that DSB-induced transcriptional silencing is also impaired in the EZH2-depleted cells (Figure 5B), suggesting that both PcG complexes are required for this activity. Moreover, we find a delay in the repair of a subset of DSBs at early time points following IR in BMI1 and EZH2-depleted cells (Figure 5C), similar to cells lacking PBAF (Figure 2), consistent with the notion that a failure to repress transcription-flanking DSBs impedes efficient repair.

Cancer-Associated Mutations of BAF180 Do Not Complement DSB Repair and Transcriptional Silencing Defects Associated with Loss of BAF180

The gene encoding BAF180 (PBRM1) is frequently mutated in cancer, and in particular, in clear cell renal cell carcinoma (ccRCC; Varela et al., 2011). We investigated whether point mutations identified in ccRCC tumor samples affected the DSB repair and DNA damage-induced transcriptional silencing functions of BAF180. We focused on missense mutations in bromodomains 2 (T232P) and 4 (M523I; corresponding to M538I in isoform 8; Figure 6A) identified in ccRCC samples (Varela et al., 2011). We recently examined these within the context of both BAF180 and its yeast homolog and found that they are stably expressed and competent for a subset of cellular functions, including transcriptional regulation, yet the mutants display genome instability, and we found a defect in sister chromatid cohesion (Brownlee et al., 2014). We introduced these mutations into our siRNA-resistant expression construct and transfected them into cells depleted for BAF180. We found that, in contrast to the WT construct, neither of the cancer-associated mutant BAF180 constructs was able to fully rescue either the DSB repair defect or the failure to arrest transcription observed in the BAF180-depleted cells (Figures 6B and 6C), raising the possibility that the function of BAF180 in repressing transcription near DSBs contributes to its tumor suppressor activity.

DISCUSSION

Here, we found that the chromatin remodeling complex PBAF is important for DNA damage-induced transcriptional silencing as well as the repair of a subset of rapidly repaired DNA DSBs. Because we can overcome the delay in repair by globally inhibiting transcription, this suggests that this delay is specifically linked to the repair of the DSBs occurring in the vicinity of actively transcribed genes. This provides evidence of a physiological consequence of loss of DNA damage-induced transcriptional repression.

In addition, these data suggest that the subset of DSBs located in chromatin-flanking transcriptionally active genes is normally repaired very rapidly—within 20 min following IR. This is perhaps not surprising, given their open chromatin configuration, which would allow for rapid detection and repair, but it may also reflect an evolutionary pressure to repair these breaks in a timely manner due to the increased vulnerability of DSBs within transcription factories to translocations (Lin et al., 2009; Mathas et al., 2009).

Our data demonstrating that a phosphomimetic (Ser-to-Glu) construct of BAF180 can partly overcome the defect in transcriptional repression due to ATM inhibition indicate that PBAF functions downstream of ATM to promote efficient H2A ubiquitination at sites of DNA DSBs. The BAF180 phosphorylation site (Ser 948 in isoform 1) is not located within any of the known domains of BAF180 (the six bromodomains, two BAH domains, or the HMG box domain). We are currently investigating the possibility that phosphorylation in this region creates a protein interaction interface that is specific to its role in this pathway.

PBAF Promotes H2AK119ub but Not H2A K13/15-Dependent Ubiquitin Chain Formation

While depletion of BAF180 resulted in impaired H2AK119ub foci following IR, we found no defect in 53BP1 or FK2 foci formation, indicating that H2A K13/15 ubiquitination and downstream signaling is not dependent on PBAF. BMI1, like PBAF, is important for global damage-induced H2AK119ub foci formation but does not affect RAP80 or FK2 foci formation (Ginjala et al., 2011), providing further evidence that these ubiquitin signaling pathways are separable and lead to distinct cellular outcomes.

There are a number of potential mechanisms by which PBAF could promote the accumulation of this modification. ATM is required for the maintenance of this modification, which is removed by USP16 when ATM is inhibited (Shanbhag et al., 2010). One way in which PBAF may facilitate the accumulation of H2AK119ub, therefore, is by preventing access of USP16 to the chromatin flanking the DSB. In support of this possibility, it was recently shown that SWI/SNF subunits (common to both BAF and PBAF complexes) preferentially localize to chromatin-containing ubiquitinated H2B (Shema-Yaacoby et al., 2013), raising the possibility that ubiquitinated H2A containing chromatin is also a preferred binding substrate. In this scenario, PBAF may rapidly bind to H2AK119ub-containing chromatin and shield it from deubiquitination by USP16.

Alternatively, it is possible that PBAF remodels the chromatin flanking the break in order to facilitate PRC2 and subsequent PRC1 activity toward their respective substrates. At promoters, PRC2 methylates H3 K27, but, consistent with one previous report (Campbell et al., 2013), we did not find an accumulation of H3K27me3 at sites of DSBs (data not shown), which may suggest that an alternative PRC2 methylation target functions to mediate PRC1 recruitment to DSBs.

(F) The formation of conjugated ubiquitin IR-induced foci (IRIF) (FK2) and 53BP1 IRIF is unaffected by siBAF180 in A549 cells exposed to 1.5 Gy IR and fixed 1 hr post-IR.
(G) Quantification of FK2 and H2A K119Ub IRIF in A549 cells exposed to 1.5 Gy IR and fixed 1 hr post-IR. Data are represented as mean ± SD.
See also Figure S5.

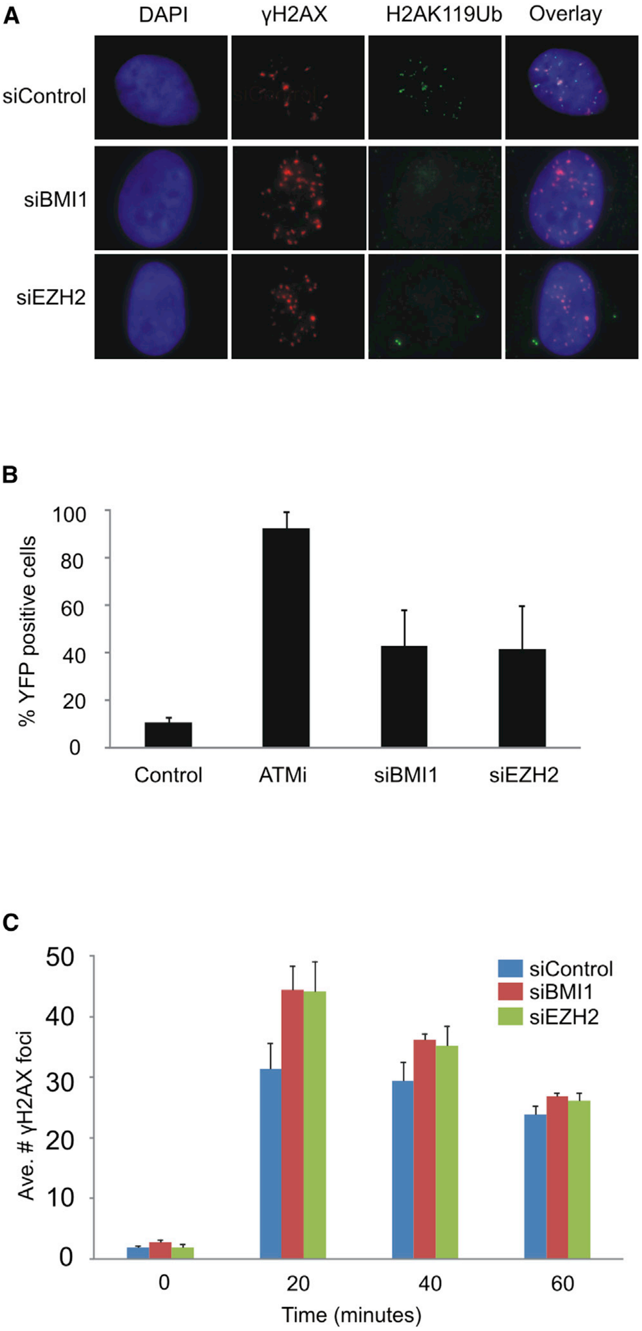

Figure 5. BMI1 and EZH2, Subunits of PRC1 and PRC2, Respectively, Are Required for DSB-Induced H2AK119ub and Transcriptional Silencing and Efficient DSB Repair at Early Time Points

(A) The formation of H2AK119ub IR-induced foci (IRIF) is defective in cells depleted for either BMI1 or EZH2 in A549 cells, 60 min following exposure to 1.5 Gy IR.

(B) Quantification of YFP-positive cells as a measure of DSB-induced transcriptional repression in U2OS reporter cells treated with the indicated siRNA. Data are represented as mean ± SD.

(C) Quantification of γH2AX foci clearance following exposure to 1.5 Gy IR in U2OS cells treated with the indicated siRNA. Data are represented as mean ± SD. Ave., average.

See also Figure S6.

We recently demonstrated that BAF180 is required for maintaining normal sister chromatid cohesion in mammalian cells (Brownlee et al., 2014). In both yeast and human cells, cohesin is additionally recruited to chromatin flanking DSBs outside of S phase, and while it is not known whether the DSB-dependent cohesin recruitment is dependent on PBAF in mammalian cells, the PBAF homolog RSC facilitates DSB-induced cohesion in yeast (Oum et al., 2011). In *Drosophila*, PRC1 interacts with cohesin, and this facilitates PRC1 binding to chromatin (Schaaf et al., 2013). One intriguing possibility therefore, is that PBAF promotes PRC1 recruitment to DSBs by regulating cohesin establishment. Finally, these mechanisms are not mutually exclusive, and it is conceivable that multiple strategies are used by PBAF to promote the accumulation of H2AK119ub and subsequent transcriptional repression at DSBs.

PBAF Activity during Tumorigenesis

The gene encoding BAF180 (PBRM1) is frequently mutated in cancer. We investigated two cancer-associated mutations of BAF180, which, we found, do not destabilize the protein (Brownlee et al., 2014). These mutations were tested within the context of the yeast homolog of BAF180 and found to be competent for a subset of cellular functions, including transcription, but in both yeast and mammalian cells, they displayed defects in maintaining genome stability and sister chromatid cohesion (Brownlee et al., 2014). Here, we found that these BAF180 mutants are also unable to restore the ability of BAF180-depleted cells to repress transcription-flanking DSBs or promote efficient repair at early time points, raising the possibility that transcriptional repression of genes flanking DSBs may be an important aspect of the ability of BAF180 to prevent tumorigenesis.

EXPERIMENTAL PROCEDURES

Laser Microirradiation

Laser microirradiation was performed in U2OS cells as described elsewhere (Rulten et al., 2011). Details of the plasmids used are provided in the Supplemental Experimental Procedures.

siRNA Transfection and IR-Induced Foci Quantification

siRNA mediated protein knockdown and γH2AX foci quantification post-IR was performed as described previously (Shibata et al., 2011). Cells were kept in G0/G1 phase by growing the cells in 5% serum. RNA interference oligo sequences are provided in the Supplemental Experimental Procedures.

Transcription Reporter Cells

The U2OS cells containing the transcription reporter construct have been described elsewhere (Shanbhag et al., 2010). These cells were transfected with 1 μg of the mCherryFokI fusion construct using *NanoJuice* transfection reagent, according to the manufacturer's protocols. Twenty-four hours later, the cells were treated with 1 μg/ml doxycycline for 3 hr. Transcription status was monitored by quantifying YFP-positive cells, and phospho-Ser2 RNAPII and H2AK119ub were monitored by immunofluorescence. Details of fluorescent signal quantification are provided in the Supplemental Experimental Procedures. A 3 hr treatment with 100 μM DRB was used to inhibit transcription in both the U20S reporter cells, as well as in A549 cells used for γH2AX foci quantification.

pEGFP-BAF180 Constructs

Complementation experiments using the pEGFP-BAF180 constructs were performed in U2OS reporter cells and in U2OS cells for transcription status and γH2AX foci quantification, respectively. Details of these constructs and

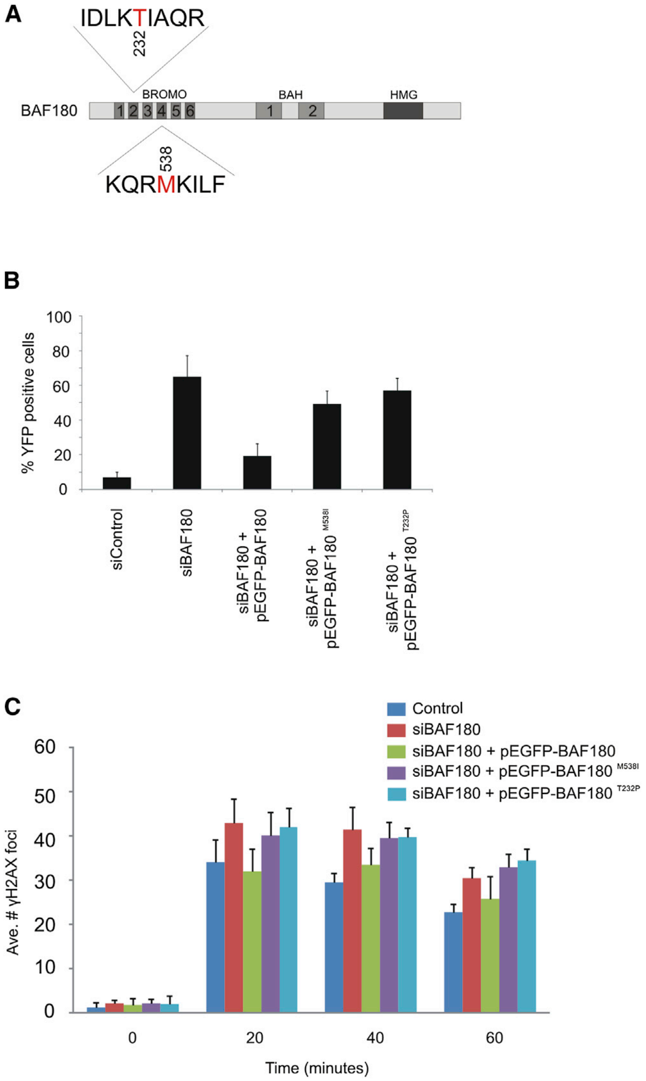

Figure 6. Cancer-Associated Mutations of BAF180 Do Not Complement DSB Repair and Transcriptional Silencing Defects Associated with Loss of BAF180

(A) Schematic representation of BAF180, showing the positions of the T232P and M538I mutations identified in ccRCC samples.

(B) Quantification of YFP-positive cells as a measure of DSB-induced transcriptional silencing in U2OS reporter cells treated with BAF180 or control siRNA and complemented with BAF180 expression constructs as indicated. Data are represented as mean ± SD.

(C) Quantification of γH2AX foci clearance following exposure to 1.5 Gy IR in U2OS cells treated with BAF180 or control siRNA and complemented with BAF180 expression constructs as indicated. Data are represented as mean ± SD.

See also Figure S1.

transfection procedures are provided in the Supplemental Experimental Procedures.

Chromosome Breakage Analysis

82-6 hTert fibroblasts were treated with nocodazole (100 ng/ml, 1 hr before IR) to prevent G2-irradiated cells from progressing into G1 during repair time. Cells were irradiated with 7 Gy. At the end of repair incubation, irradiated cells were mixed at a ratio of 1:1 with mitotic HeLa cells (enriched by 200 ng/ml colcemid for 20 hr). Polyethylene-glycol-mediated cell fusion and chromosome preparation were performed as described elsewhere (Mosesso et al., 1999).

SUPPLEMENTAL INFORMATION

Supplemental Information includes Supplemental Experimental Procedures and six figures and can be found with this article online at http://dx.doi.org/10.1016/j.molcel.2014.06.028.

ACKNOWLEDGMENTS

We thank Sarah Pinder for technical assistance and Roger Greenberg for providing cell lines and reagents. This study was supported by the Medical Research Council (P.A.J. and J.A.D.) and Cancer Research UK (J.A.D.).

Received: November 19, 2013
Revised: May 1, 2014
Accepted: June 19, 2014
Published: July 24, 2014

REFERENCES

Brownlee, P.M., Chambers, A.L., Cloney, R., Bianchi, A., and Downs, J.A. (2014). BAF180 promotes cohesion and prevents genome instability and aneuploidy. Cell Reports *6*, 973–981.

Campbell, S., Ismail, I.H., Young, L.C., Poirier, G.G., and Hendzel, M.J. (2013). Polycomb repressive complex 2 contributes to DNA double-strand break repair. Cell Cycle *12*, 2675–2683.

Cao, R., Tsukada, Y., and Zhang, Y. (2005). Role of Bmi-1 and Ring1A in H2A ubiquitylation and Hox gene silencing. Mol. Cell *20*, 845–854.

Chambers, A.L., and Downs, J.A. (2012). The RSC and INO80 chromatin-remodeling complexes in DNA double-strand break repair. Prog. Mol. Biol. Transl. Sci. *110*, 229–261.

Chou, D.M., Adamson, B., Dephoure, N.E., Tan, X., Nottke, A.C., Hurov, K.E., Gygi, S.P., Colaiácovo, M.P., and Elledge, S.J. (2010). A chromatin localization screen reveals poly (ADP ribose)-regulated recruitment of the repressive polycomb and NuRD complexes to sites of DNA damage. Proc. Natl. Acad. Sci. USA *107*, 18475–18480.

Fradet-Turcotte, A., Canny, M.D., Escribano-Díaz, C., Orthwein, A., Leung, C.C., Huang, H., Landry, M.C., Kitevski-LeBlanc, J., Noordermeer, S.M., Sicheri, F., and Durocher, D. (2013). 53BP1 is a reader of the DNA-damage-induced H2A Lys 15 ubiquitin mark. Nature *499*, 50–54.

Francia, S., Michelini, F., Saxena, A., Tang, D., de Hoon, M., Anelli, V., Mione, M., Carninci, P., and d'Adda di Fagagna, F. (2012). Site-specific DICER and DROSHA RNA products control the DNA-damage response. Nature *488*, 231–235.

Ginjala, V., Nacerddine, K., Kulkarni, A., Oza, J., Hill, S.J., Yao, M., Citterio, E., van Lohuizen, M., and Ganesan, S. (2011). BMI1 is recruited to DNA breaks and contributes to DNA damage-induced H2A ubiquitination and repair. Mol. Cell. Biol. *31*, 1972–1982.

Goodarzi, A.A., and Jeggo, P.A. (2013). The repair and signaling responses to DNA double-strand breaks. Adv. Genet. *82*, 1–45.

Khavari, P.A., Peterson, C.L., Tamkun, J.W., Mendel, D.B., and Crabtree, G.R. (1993). BRG1 contains a conserved domain of the SWI2/SNF2 family necessary for normal mitotic growth and transcription. Nature *366*, 170–174.

Lin, C., Yang, L., Tanasa, B., Hutt, K., Ju, B.G., Ohgi, K., Zhang, J., Rose, D.W., Fu, X.D., Glass, C.K., and Rosenfeld, M.G. (2009). Nuclear receptor-induced chromosomal proximity and DNA breaks underlie specific translocations in cancer. Cell *139*, 1069–1083.

Mathas, S., Kreher, S., Meaburn, K.J., Jöhrens, K., Lamprecht, B., Assaf, C., Sterry, W., Kadin, M.E., Daibata, M., Joos, S., et al. (2009). Gene deregulation and spatial genome reorganization near breakpoints prior to formation of

translocations in anaplastic large cell lymphoma. Proc. Natl. Acad. Sci. USA *106*, 5831–5836.

Matsuoka, S., Ballif, B.A., Smogorzewska, A., McDonald, E.R., 3rd, Hurov, K.E., Luo, J., Bakalarski, C.E., Zhao, Z., Solimini, N., Lerenthal, Y., et al. (2007). ATM and ATR substrate analysis reveals extensive protein networks responsive to DNA damage. Science *316*, 1160–1166.

Mattiroli, F., Vissers, J.H., van Dijk, W.J., Ikpa, P., Citterio, E., Vermeulen, W., Marteijn, J.A., and Sixma, T.K. (2012). RNF168 ubiquitinates K13-15 on H2A/H2AX to drive DNA damage signaling. Cell *150*, 1182–1195.

Mosesso, P., Fonti, E., Bassi, L., Lorenti Garcia, C., and Palitti, F. (1999). The involvement of chromatin condensation in camptothecin-induced chromosome breaks in G0 human lymphocytes. Mutagenesis *14*, 103–105.

Noon, A.T., Shibata, A., Rief, N., Löbrich, M., Stewart, G.S., Jeggo, P.A., and Goodarzi, A.A. (2010). 53BP1-dependent robust localized KAP-1 phosphorylation is essential for heterochromatic DNA double-strand break repair. Nat. Cell Biol. *12*, 177–184.

Oum, J.H., Seong, C., Kwon, Y., Ji, J.H., Sid, A., Ramakrishnan, S., Ira, G., Malkova, A., Sung, P., Lee, S.E., and Shim, E.Y. (2011). RSC facilitates Rad59-dependent homologous recombination between sister chromatids by promoting cohesin loading at DNA double-strand breaks. Mol. Cell. Biol. *31*, 3924–3937.

Papamichos-Chronakis, M., and Peterson, C.L. (2013). Chromatin and the genome integrity network. Nat. Rev. Genet. *14*, 62–75.

Rulten, S.L., Fisher, A.E., Robert, I., Zuma, M.C., Rouleau, M., Ju, L., Poirier, G., Reina-San-Martin, B., and Caldecott, K.W. (2011). PARP-3 and APLF function together to accelerate nonhomologous end-joining. Mol. Cell *41*, 33–45.

Schaaf, C.A., Misulovin, Z., Gause, M., Koenig, A., Gohara, D.W., Watson, A., and Dorsett, D. (2013). Cohesin and polycomb proteins functionally interact to control transcription at silenced and active genes. PLoS Genet. *9*, e1003560.

Shain, A.H., and Pollack, J.R. (2013). The spectrum of SWI/SNF mutations, ubiquitous in human cancers. PLoS ONE *8*, e55119.

Shanbhag, N.M., Rafalska-Metcalf, I.U., Balane-Bolivar, C., Janicki, S.M., and Greenberg, R.A. (2010). ATM-dependent chromatin changes silence transcription in cis to DNA double-strand breaks. Cell *141*, 970–981.

Shema-Yaacoby, E., Nikolov, M., Haj-Yahya, M., Siman, P., Allemand, E., Yamaguchi, Y., Muchardt, C., Urlaub, H., Brik, A., Oren, M., and Fischle, W. (2013). Systematic identification of proteins binding to chromatin-embedded ubiquitylated H2B reveals recruitment of SWI/SNF to regulate transcription. Cell Reports *4*, 601–608.

Shibata, A., Conrad, S., Birraux, J., Geuting, V., Barton, O., Ismail, A., Kakarougkas, A., Meek, K., Taucher-Scholz, G., Löbrich, M., and Jeggo, P.A. (2011). Factors determining DNA double-strand break repair pathway choice in G2 phase. EMBO J. *30*, 1079–1092.

Stiff, T., O'Driscoll, M., Rief, N., Iwabuchi, K., Löbrich, M., and Jeggo, P.A. (2004). ATM and DNA-PK function redundantly to phosphorylate H2AX after exposure to ionizing radiation. Cancer Res. *64*, 2390–2396.

Varela, I., Tarpey, P., Raine, K., Huang, D., Ong, C.K., Stephens, P., Davies, H., Jones, D., Lin, M.L., Teague, J., et al. (2011). Exome sequencing identifies frequent mutation of the SWI/SNF complex gene PBRM1 in renal carcinoma. Nature *469*, 539–542.

Vissers, J.H., van Lohuizen, M., and Citterio, E. (2012). The emerging role of Polycomb repressors in the response to DNA damage. J. Cell Sci. *125*, 3939–3948.

Wei, W., Ba, Z., Gao, M., Wu, Y., Ma, Y., Amiard, S., White, C.I., Rendtlew Danielsen, J.M., Yang, Y.G., and Qi, Y. (2012). A role for small RNAs in DNA double-strand break repair. Cell *149*, 101–112.

Molecular Cell, Volume *55*

Supplemental Information

Requirement for PBAF in Transcriptional Repression and Repair at DNA Breaks in Actively Transcribed Regions of Chromatin

Andreas Kakarougkas, Amani Ismail, Anna L. Chambers, Enriqueta Riballo, Alex D. Herbert,
Julia Künzel, Markus Löbrich, Penny A. Jeggo, and Jessica A. Downs

SUPPLEMENTAL INFORMATION

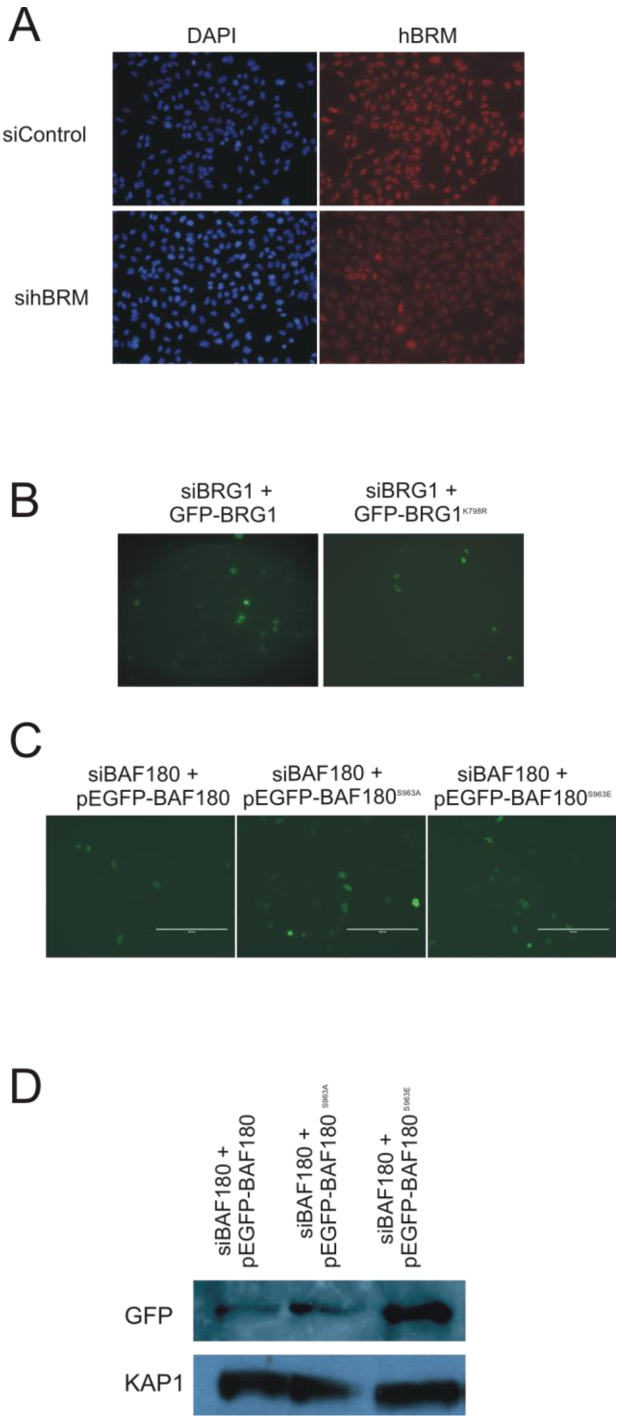

Figure S1, related to Figures 1 and 3. Expression of GFP tagged BAF180 and BRG1 constructs. (A) Knockdown efficiency of hBRM siRNA was assessed by immunofluorescence in A549 cells. (B) Immunofluorescence images showing GFP expression of WT and ATPase mutated GFP-BRG1. Endogenous BRG1 was depleted using siRNA and then the cells were transfected with the siRNA resisant GFP-BRG1 constructs. (C) Immunofluorescence images showing GFP expression of WT, phosphomutant (S963A) and phosphomimic (S963E) GFP-BAF180. Endogenous BAF180 was depleted using siRNA and then the cells were transfected with the siRNA resistant GFP-BAF180 constructs. (D) Expression of the GFP-BAF180 constructs assessed by western blotting using a GFP antibody. KAP1 was used as a loading control.

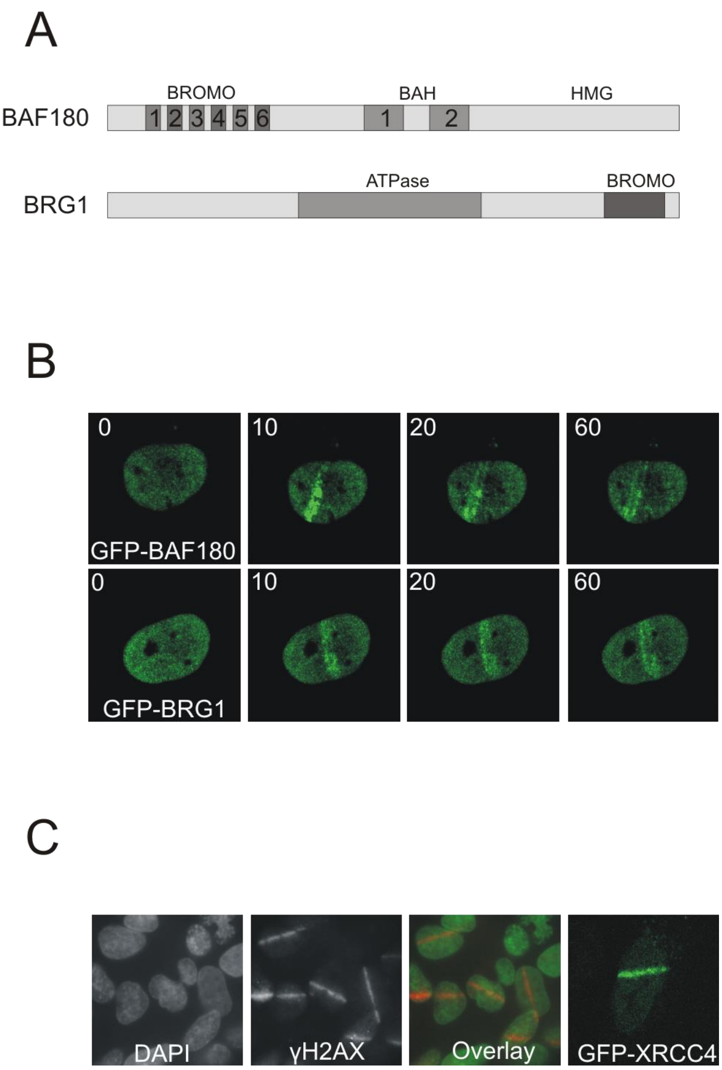

Figure S2, related to Figure 2. BAF180 and BRG1 localise to sites of DNA damage. (A) Schematic representation of BAF180 and BRG1. (B) Localised DNA damage was induced by laser irradiation in cells transfected with EGFP-tagged BAF180 or GFP-tagged BRG1. Representative images up to 60 seconds post laser irradiation. (C) Representative images of γH2AX and GFP-tagged XRCC4 recruitment to sites of laser irradiation, 60 seconds post damage induction.

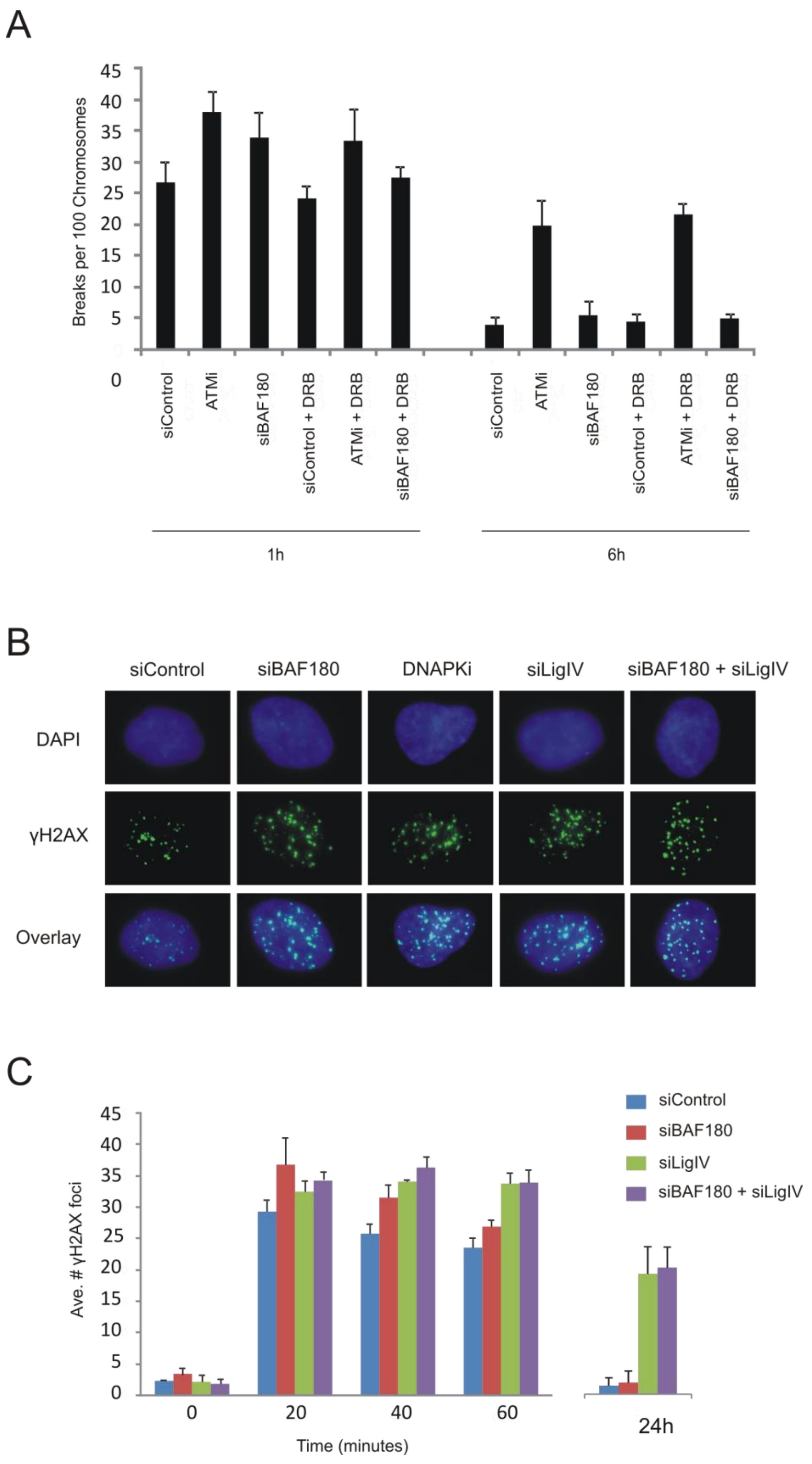

Figure S3, related to Figure 2. BAF180 promotes non-homologous end joining (NHEJ). (A) PCC breakage analysis following 2 Gy IR in HeLa cells treated with the indicated siRNA. Data are represented as mean +/- SD**.** (B) Quantification of γH2AX foci clearance following exposure to 1.5 Gy IR in A549 cells treated with the indicated siRNA. Data are represented as mean +/- SD (C) Representative IF images of A549 cells treated with the indicated siRNA, 40m following exposure to 1.5 Gy IR.

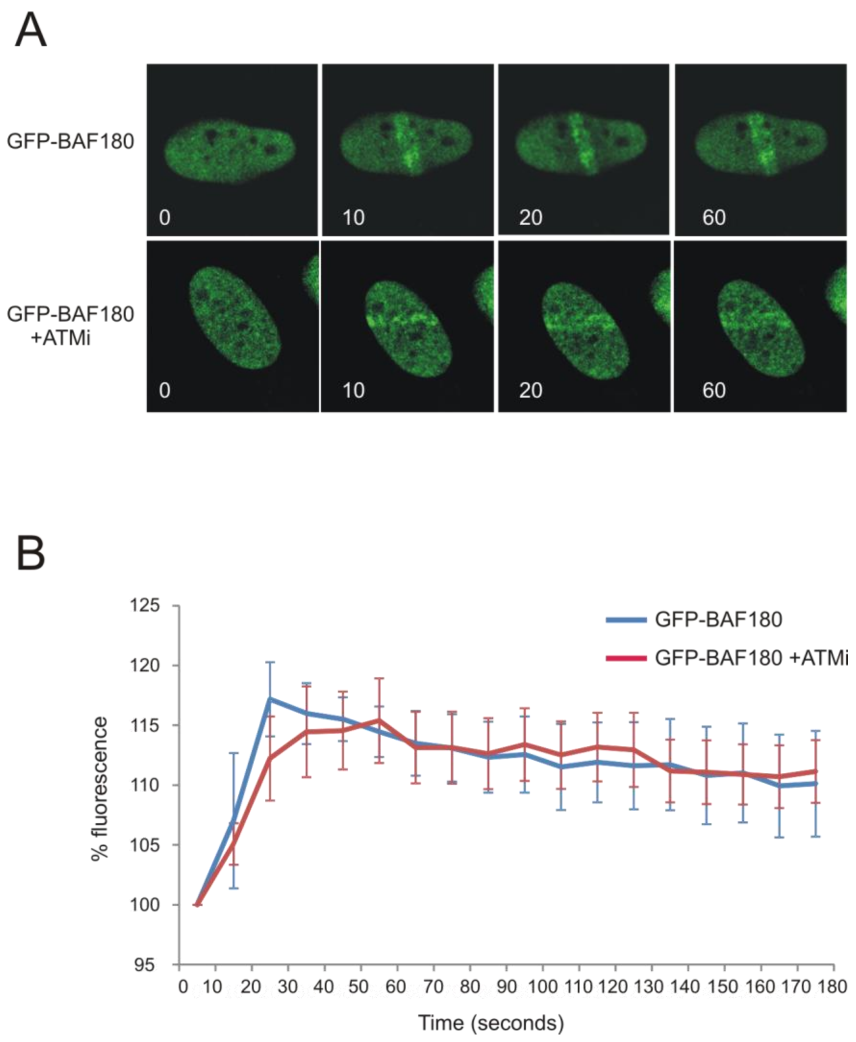

Figure S4, related to Figure 3. BAF180 recruitment to laser induced DNA damage is ATM independent. (A) Localised DNA damage was induced by laser irradiation in cells transfected with GFP-tagged BAF180. In ATMi treated cells, the inhibitor was added 1 hour prior to irradiation. Representative images up to 60 seconds post laser irradiation are shown. (B) Quantification of GFP signal intensity along the laser path in ATMi treated and untreated GFP-BAF180 expressing cells. GFP-signal intensity was monitored for 3 minutes post irradiation. Data are represented as mean +/- SD**.**

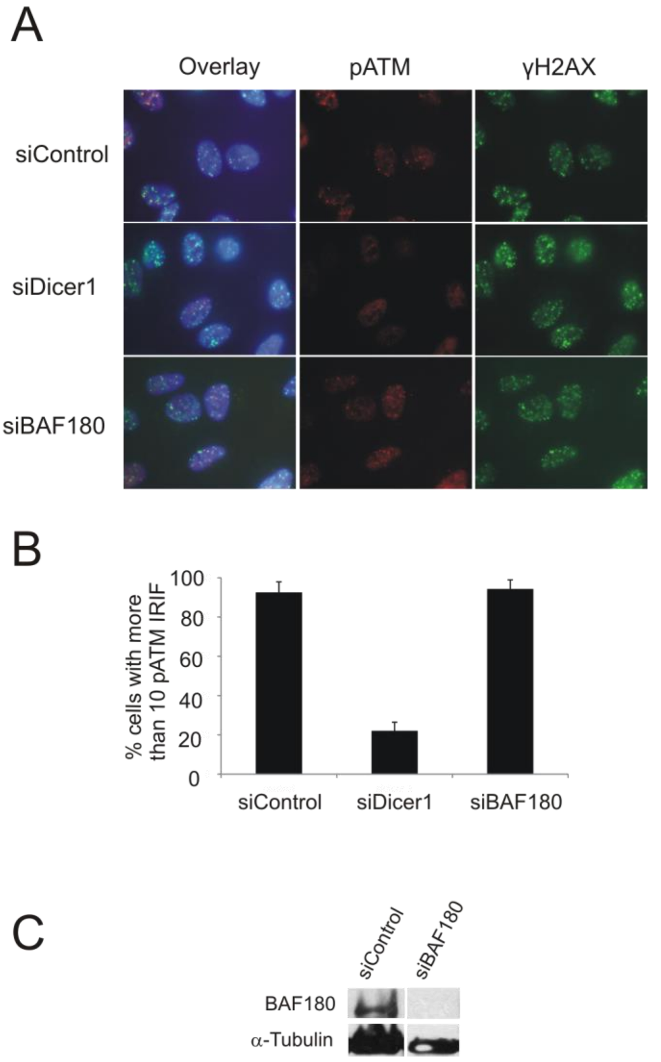

Figure S5, related to Figure 4. BAF180 functions downstream of ATM and is not required for pATM IRIF. (A) pATM and γH2AX IRIF 1 hour after 3 Gy IR in A549 cells treated with the indicated siRNA. (B) Quantification of pATM IRIF. Data are represented as mean +/- SD**.** (C) Western blot analysis of siRNA knockdown efficiency of BAF180 in A549 cells. Tubulin was used as a loading control.

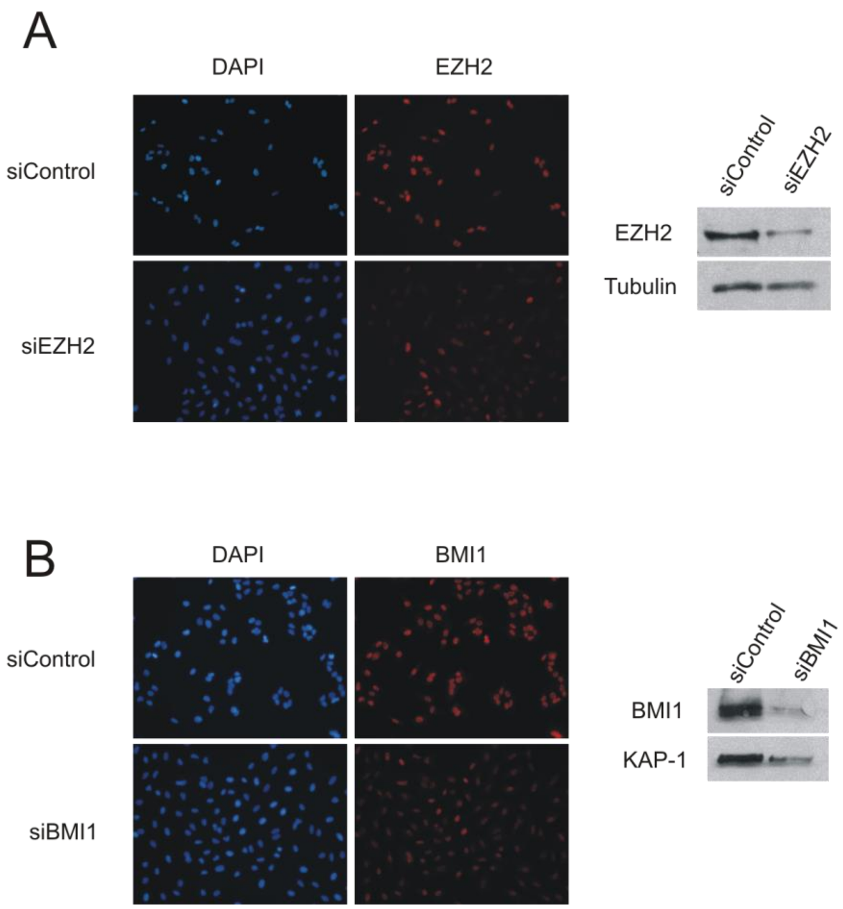

Figure S6, related to Figure 5. Knockdown efficiency of BMI1 and EZH2 siRNA. (A) Knockdown efficiency of BMI1 siRNA was assessed by immunofluorescence and western blotting in A549 cells. KAP1 was used as a loading control. (B) Knockdown efficiency of EZH2 siRNA was assessed by immunofluorescence and western blotting in A549 cells. Tubulin was used as a loading control.

SUPPLEMENTAL EXPERIMENTAL PROCEDURES

Cell Culture and irradiation

A549, HeLa and U2OS cells were cultured in DMEM (Gibco) with 10% FCS, L-glutamine, penicillin and streptomycin (Gibco) at 37°C in a humidified 95% air and 5% CO^2 atmosphere. Cells were irradiated by exposure to a ^{137}Cs source. U2OS reporter cells were cultured in DMEM (Gibco) with 10% Tet-System approved FBS (Clontech), L-glutamine, penicillin and streptomycin (Gibco), 200mg/ml G418 and 100mg/ml Hygromycin B, at 37°C in a humidified 95% air and 5% CO2 atmosphere.

Premature chromosome condensation (PCC) breakage analysis.

PCC analysis was carried out as described previously (Deckbar et al., 2007). Briefly, irradiated (2 Gy) HeLa cells were treated at the indicated time points with 50 ng/ml Calyculin-A for 0.5h to induce PCC in G2-phase cells. Cells were then harvested, fixed, and processed for chromosome break analysis. Chromosome breaks were scored per 100 chromosomes counted. All results represent the mean +/- SD of three experiments.

Laser microirradiation.

Exponentially growing human U2OS cells were plated onto 35mm glass-bottom dishes (MatTek) and transfected with the pEGFP-BAF180, GFP-XRCC4 or GFP-BRG1 constructs using NanoJuice according to the manufacturers protocol. The cells were allowed to express the construct for 24h and were then incubated with 10 µg/ml Hoechst 3458 for 1 h at 37 °C before irradiation. The microscope system used was an Intelligent Imaging Innovations spinning disk confocal with a Yokogawa CSU-X1 on an Olympus IX-71. GFP positive cells were irradiated with a 100mW, 405nm ultraviolet laser set at 20% power and channelled through a 60x objective. The UV laser was focused to an area of approximately 12 µm*0.1µm through the cell nuclei, and images were captured at 10s intervals following laser damage for a total time of 180s. Images generated were acquired on a Photometrics Evolve 512x512 EMCCD using Slidebook 5.5 software. Where indicated, 10 µMATMi (Tocris Bioscience) was added 1h prior to irradiation. In protein recruitment experiments, signal intensity was quantified along the laser path, using Slidebook 5.5 software, in a minimum of 20 cells and error bars represent the SD between three independent experiments.

Small interfering RNA (siRNA) knockdown conditions

siRNA-mediated knockdown was achieved using HiPerFect Transfection Reagent (Qiagen, Hilden, Germany) following the manufacturer’s instructions.siRNA duplexes were

transfected into 2 x 10^5 of logarithmically growing cells per condition. Cells were harvested 24h later, re-transfected with siRNA and then seeded and grown for 48 hours. The BAF180 siRNA oligonucleotide is 5′-GAGAAATCTTGAGACAGCCAAGAAA-3′ (Invitrogen Stealth RNAi). The BRG1 siRNA is a pool of 3 target specific siRNAs from Santa Cruz. For BRG1 complementation experiments the siRNA oligonucleotide used is 5'-CGCGGCACATCATTGAGAATGCCAA-3'. The RNF8 siRNA was described previously (Mailand et al., 2007). The BMI1 and EZH2 siRNA oligonucleotides are from the Dharmacon smartpool.

Antibodies

The primary antibodies used were: γH2AX (Upstate Technology) at 1:800 for IF, anti-phospho Ser2 RNAPII (Abcam) at 1:500 for IF, Anti-monoubiquityl-Histone H2A (Lys119) (Upstate Technology) at 1:500 for (IF), FK2 (Enzo) at 1:400 for IF, BAF180 (Bethyl) at 1:2000 for WB, α-Tubulin (Abcam) at 1:5000 for WB, KAP1 (Abcam) 1:1000 for WB, EZH2 (New England Bioloabs) 1:400 for IR and 1:1000 for WB, BMI1 (Cambridge Bioscience) 1:400 for IF and 1:1000 for WB.

The secondary antibodies used were: FITC (Sigma Aldrich) at 1:200 for IF, CY3 (Sigma Aldrich) at 1:200 for IF, Alexa647 (Invitrogen) at 1:400 for IF, Polyclonal Goat Anti-Rabbit Immunoglobulins/HRP at 1:2000 for WB, Polyclonal Rabbit Anti-Mouse Immunoglobulins/HRP at 1:2000 for WB.

Immunofluorescence

Cells plated on glass slides were fixed for 10 min with fixative (3% (w/v) PFA, 2% (w/v) sucrose, 1X PBS) and permeabilized for 1 min with 0.2 % Triton X-100 in PBS. When staining for H2AK119ub, pre-extraction was performed by treatment with 0.2% Triton X-100 in PBS for 0.5-1 min prior to PFA fixation. Cells were rinsed with PBS and incubated with primary antibody diluted in PBS + 2% (w/v) BSA for 1 h at room temperature (RT). Cells were washed three times, incubated with secondary antibody (diluted in PBS + 2% (w/v) BSA) for 30 min at RT in the dark, incubated with 4′,6-diamidino-2-phenylindole (DAPI) where indicated for 10 min, and washed three times with PBS. Slides were mounted using Vectashield and visualised/analysed using a Nikon-e400 microscope and imaged using an Applied Precision® Delta Vision® RT Olympus IX70 deconvolution microscope and softWoRx® Suite software. For γH2AX foci quantification, a minimum of 30 cells was scored blindly and error bars represent the SD between three independent experiments. In complementation experiments, IRIF were only scored in GFP expressing cells.

H2AK119ub and FK2 immunofluorescence signal quantification was carried out using ImageJ. The "Spot Analyser" plugin and the associated script are freely available at http://www.sussex.ac.uk/gdsc/intranet/microscopy/imagej/utility. Here, cell nuclei were detected using Otsu's thresholding method (Otsu, 1979). The plugin smooths the image using a Gaussian blur to aid spot detection on noisy data. In this case, panuclear YFP signal was used to detect cell nuclei in the U2OS cells containing the transcriptional reporter system. Next, the plugin was used to detect the reporter site (mCherry expression) within the nucleus by searching for intensity maxima above the background level. The background is set using the mean intensity within the nuclei.The maxima are enlarged using a watershed algorithm of steepest descent. The final maxima region is then pruned back to a fraction of its height above the background. Subsequently, fluorescence intensity measurements inside and outside the maxima were obtained for H2AK119ub and FK2, and expressed as a ratio. In summary, the following parameters were used: Threshold method- Otsu, Blur- 3, Minimum particle size- 2000, Number of peaks- 1, Fraction- 0.9.   A minimum of 10 cells were analysed per condition from three independent experiments. The data are normalised to the measurements in control cells. Error bars represent SD.

Quantification of transcription status in U2OS transcription reporter cells.

Transcription status was monitored in U2OS reporter cells following treatment with the indicated siRNAs and following transient expression of the indicated constructs. U2OS reporter cells were plated on glass coverslips and transfected with 1µg mCherryFokI using *NanoJuice*® transfection reagent. 24h later, 1µg/ml doxycycline was added for three hours. Where indicated, 10 µM ATMi (Tocris Bioscience) was added to the cells 60 minutes prior to the addition of doxycycline. Where indicated, 100 µM DRB for was added for 3 hr prior to the addition of doxycycline. Following doxycycline treatment, cells were fixed and mounted as described above. Transcription status was monitored using a Nikon-e400 microscope by quantifying YFP positive cells at the transcription reporter site, located by mCherry signal. A minimum of 100 cells were analysed blindly and error bars represent the SD between three independent experiments.

Plasmids and Constructs

The mCherryFokI construct was described previously (Shanbhag et al., 2010). BAF180 complete cDNA (clone MGC:156155, IMAGE:40082629) was purchased from Source BioScience and cloned into *Hind*II/*Kpn*I sites of pEGFP-C3 (Clontech). This clone corresponds to isoform 8 and contains a N122S substitution relative to the published sequence. To generate siRNA-resistant BAF180 expression constructs, T285C, G291A and C297T silent point mutations were introduced into the BAF180 cDNA of pEGFP-

BAF180 by site directed mutagenesis (QuickChange, Agilent), using the oligonucleotides 5′- GTGGGGACAGAATGGAGAAACCTTGAAACAGCTAAGAAAGCAGAATATGAAG-3′ (forward) and 5'-CTTCATATTCTGCTTTCTTAGCTGTTTCAAGG TTTCTCCATTCTGTCCCCAC-3' (reverse) to create pEGFP-BAF180r. The pEGFP-BAF180r^{S963A}, and pEGFP-BAF180r^{S963E} constructs were generated by site directed mutagenesis (QuickChange, Agilent). The pEGFP-BAF180r^{T232P}and pEGFP-BAF180r^{M538I} constructs were described previously (Brownlee et al., 2014).

GFP-tagged, human BRG1 cDNA ORF clone (NM_001128849.1) was purchased in the pCMV6-AC-His vector from Origene. To generate siRNA-resistant BRG1 expression constructs, G2186A, C2192A, G2198A and C2204A silent point mutations were introduced into the cDNA of GFP-BRG1 by site directed mutagenesis (QuikChange Lightning Multi Site-Directed Mutagenesis, Agilent) using the oligonucleotide 5'-CTGAGGTGGACGCGCGACACATAATTGAAAATGCGAAGCAAGATGTCGATG-3'. The K798R mutation in the siRNA resistant GFP-BRG1 expression construct was generated using the oligonucleotide 5'-ATGGGCCTGGGGAGGACCATCCAGACC-3'.

SUPPLEMENTAL REFERENCES

Brownlee, P.M., Chambers, A.L., Cloney, R., Bianchi, A., and Downs, J.A. (2014). BAF180 Promotes Cohesion and Prevents Genome Instability and Aneuploidy. Cell reports *6*, 973-981.

Deckbar, D., Birraux, J., Krempler, A., Tchouandong, L., Beucher, A., Walker, S., Stiff, T., Jeggo, P., and Lobrich, M. (2007). Chromosome breakage after G2 checkpoint release. J Cell Biol *176*, 749-755.

Mailand, N., Bekker-Jensen, S., Faustrup, H., Melander, F., Bartek, J., Lukas, C., and Lukas, J. (2007). RNF8 Ubiquitylates Histones at DNA Double-Strand Breaks and Promotes Assembly of Repair Proteins. Cell *131*, 887-900.

Otsu, (1979). A Threshold Selection Method from Gray-Level Histograms.IEEE Transactions *9*, 62-66.

Shanbhag, N.M., Rafalska-Metcalf, I.U., Balane-Bolivar, C., Janicki, S.M., and Greenberg, R.A. (2010). ATM-dependent chromatin changes silence transcription in cis to DNA double-strand breaks. Cell *141*, 970-981.
